# Histone acetylation-related IncRNA: Potential biomarkers for predicting prognosis and immune response in lung adenocarcinoma, and distinguishing hot and cold tumours

**DOI:** 10.3389/fimmu.2023.1139599

**Published:** 2023-03-17

**Authors:** Rumeng Li, Tingting Qiu, Qiangqiang Zhou, Fajian He, Chen Jie, Xinyu Zheng, Zeguang Lu, Qiuji Wu, Conghua Xie

**Affiliations:** ^1^ Department of Radiation and Medical Oncology, Zhongnan Hospital of Wuhan University, Wuhan, China; ^2^ Jiangxi Cancer Hospital, The Second Affiliated Hospital of Nanchang Medical College, Jiangxi Clinical Research Center for Cancer, Nanchang, China; ^3^ Department of Neurosurgery, Zhongnan Hospital of Wuhan University, Wuhan, China; ^4^ Department of Gastrointestinal Surgery, Zhongnan Hospital of Wuhan University, Wuhan, China; ^5^ The Second Clinical College of Guangzhou Medical University, Guangzhou, China; ^6^ Hubei Key Laboratory of Tumor Biological Behaviors, Zhongnan Hospital of Wuhan University, Wuhan, China

**Keywords:** long noncoding RNAs, lung adenocarcinoma, biomarker, histone acetylation, drug sensitivity, epigenetics

## Abstract

**Background:**

Histone acetylation-related lncRNAs (HARlncRNAs) play significant roles in various cancers, but their impact on lung adenocarcinoma (LUAD) remains unclear. This study aimed to develop a new HARlncRNA-based prognostic model for LUAD and to explore its potential biological mechanisms.

**Methods:**

We identified 77 histone acetylation genes based on previous studies. HARlncRNAs related to prognosis were screened by co-expression, univariate and multivariate analyses, and least absolute shrinkage selection operator regression (LASSO). Afterward, a prognostic model was established based on the screened HARlncRNAs. We analysed the relationship between the model and immune cell infiltration characteristics, immune checkpoint molecule expression, drug sensitivity, and tumour mutational burden (TMB). Finally, the entire sample was divided into three clusters to further distinguish between hot and cold tumours.

**Results:**

A seven-HARlncRNA-based prognostic model was established for LUAD. The area under the curve (AUC) of the risk score was the highest among all the analysed prognostic factors, indicating the accuracy and robustness of the model. The patients in the high-risk group were predicted to be more sensitive to chemotherapeutic, targeted, and immunotherapeutic drugs. It was worth noting that clusters could effectively identify hot and cold tumours. In our study, clusters 1 and 3 were considered hot tumours that were more sensitive to immunotherapy drugs.

**Conclusion:**

We developed a risk-scoring model based on seven prognostic HARlncRNAs that promises to be a new tool for evaluating the prognosis and efficacy of immunotherapy in patients with LUAD.

## 1 Introduction

Lung cancer is the leading cause of cancer-related death and morbidity worldwide (1, 2). Most patients with lung cancer have developed advanced disease at the time they see a doctor. The 5-year survival rate for lung cancer patients is < 30% despite advances in diagnosis and treatment (3). Therefore, it is important to identify individualised biomarkers related to lung cancer to improve the precision of treatment and prognosis.

The incidence of cancer continues to rise and its development is inseparable from gene mutations and epigenetics (4). Histone modifications mainly include methylation, acetylation, phosphorylation, and ubiquitination (5). These modifications can be combined to form a ‘ histone code ‘ that regulates gene expression (6). During histone acetylation, an acetyl group is covalently added to the amino group of the lysine residue at the tail of the histone (7). Maintaining the balance of histone acetylation modification is essential for regulating gene expression and maintaining cell homeostasis. The removal of acetyl groups from histones leads to chromatin compression, thereby inhibiting the transcription of corresponding genes (8). Lung cancer cells increased acetylation of H4K5/H4K8 and decreased acetylation of H4K12/H4K16 compared to normal lung epithelial cells (9). Moreover, *HDAC2* and *TRIM24* were found to be overexpressed in lung cancer (10, 11). Zhou et al. found that *KAT2B* is mainly associated with antigen processing and presentation, immune cell regulation, and IFN-γ response, and its expression is associated with poor prognosis of LUAD (12). Therefore, the investigation of histone acetylation is of great significance for LUAD.

Longnon-codingRNAs (LncRNAs) do not directly encode proteins but can affect the expression of target genes through epigenetic regulation of gene expression processes, such as transcription and translation. In addition, lncRNAs can not only regulate the growth and differentiation of cancer cells (13–17), but also the ability of cancer cells to invade other organs and participate in the mechanism of drug resistance by cancer cells (18). The lncRNA HULC showed a cancer-promoting effect on glioblastoma, gastric cancer, and pancreatic cancer (19, 20). LINC00973 is involved in chemoresistance in colon cancer, whereas LINC00092 promotes ovarian cancer progression by driving glycolysis through tumour-associated fibroblasts (21, 22). Using microarray analysis, Wang et al. found that lncRNAs can distinguish LUAD from normal tissues and have high sensitivity and specificity, suggesting that abnormally expressed lncRNAs are expected to become signature biomarkers for LUAD diagnosis (23). Qiu and Luo et al. found that lncRNAs CCAT2 and CARLo-5 were elevated in LUAD and associated with poor prognosis (24, 25). Ji et al. found that MALAT1, a new non-coding RNA, can predict the metastasis and survival of early non-small cell lung cancer (NSCLC) (26). Although many lncRNAs have been shown to have important prognostic value in lung cancer, their roles have not been completely elucidated (27). In addition to affecting the tumour cells themselves, lncRNAs can also mediate the interaction between tumour and immune cells (28), thereby affecting the tumour microenvironment (TME) (29). In recent years, breakthroughs in tumour immunotherapy have extensively expanded the field of tumour therapy. However, drug resistance significantly limits its benefits to patients (30, 31). Tumour-associated long noncoding RNAs inhibit antigen presentation and immune cell infiltration, thereby reducing the effectiveness of immune checkpoint therapy (32, 33). For example, MALAT1 was first identified in lung cancer patients. It can not only regulate the expression of PL-L1 (34), but also regulate the infiltration of CD8+ T cells and myeloid-derived suppressor cells (MDSCs) (35). LINC00473 increased the expression of PD-L1 and its receptor PD-1 in pancreatic cancer cells by sponging miR-195-5p, thereby inhibiting the activation of CD8+ T cells (36). Given the important role of lncRNAs in tumour and immunotherapy resistance, the combined use of targeted lncRNA and chemotherapy drugs or immunotherapy may be an effective strategy for cancer treatment (28, 37). Studies have demonstrated that lncRNAs promote cancer progression by acetylating post-translational modifications of key metabolism-related proteins (38, 39). Although histone acetylation-related lncRNAs (HARlncRNAs) cannot directly encode proteins, they can transmit downstream information and regulate the expression of histone acetylation-related genes. However, the function of HARlncRNAs in LUAD remains unclear.

In the present study, we investigated the correlation between HARlncRNAs and LUAD prognosis. We screened independent prognostic HARlncRNAs to construct a risk-scoring model. The model was verified using receiver operating characteristic (ROC) analysis and a nomogram. We further examined the relationship between the risk model and immune infiltration, TMB, chemotherapy, targeted therapy, and immunotherapy sensitivity. Finally, the entire sample was divided into three clusters to further distinguish between hot and cold tumours. These findings may provide new prognostic tools and potential biomarkers for LUAD.

## 2 Methods

### 2.1 Data sources for research

The gene expression profiles, somatic mutation data, and clinical data of patients with LUAD were obtained from the TCGA-LUAD database. We included patients who met the pathological diagnosis of lung adenocarcinoma and had complete follow-up and clinical information. In addition, we excluded patients with a survival time < 30 days. Basic patient information is provided in **Supplementary Table 1**.

### 2.2 Modelling based on 7 lncRNAs

We summarised 77 previously identified histone acetylation-related genes (40). Afterward, we used the limma package in R to screen for differentially expressed genes (DEGs) in LUAD. Genes without corresponding lncRNAs in TCGA-LUAD were excluded from our study. A total of 4241 HARlncRNAs were screened according to a correlation coefficient > 0.4, p< 0.05.

After univariate Cox regression analysis in the TCGA cohort, we performed minimum absolute shrinkage, LASSO regression, and multivariate stepwise Cox regression analyses, and finally obtained 7 histones acetylated lncRNAs in the risk characteristics. An equal ratio of 1:1 was used to divide the dataset into training and validation datasets.

Based on the median risk scores, the training and validation groups were divided into high- and low-risk groups, respectively. Additionally, we performed principal component analysis (PCA) to verify the independent prognostic ability of the model. The C-index is mainly used to evaluate the prediction accuracy of the established model.

### 2.3 Risk score and clinicopathological features

To determine the stability of the constructed risk score in predicting survival outcomes in clinical features, including age, sex, and pathological stage, we plotted the Kaplan-Meier curve for different groups.

### 2.4 Tool to assess the prognosis of patients

A nomogram is a common tool for evaluating prognosis. It integrates various clinicopathological features that are associated with prognosis. The nomogram was built using the R package ‘rms’.

### 2.5 Functional pathways of DEGs

To understand the mechanism and potential biological functions of DEGs in the high- and low-risk groups in LUAD, a cluster profile software package was used for Gene Ontology (GO) enrichment analysis in R software. P< 0.05 was considered statistically significant. The results were plotted using the ggplot2 software package.

### 2.6 Immune profile analysis and immune checkpoint inhibitor treatment response prediction 

First, we analysed the expression differences of immune checkpoint-related genes in the two groups of patients. Afterward, we analysed the immune and matrix scores of the TME using an estimate algorithm (41). Immune cells and enriched immune-related functions of the two groups were analysed using single-sample gene-set enrichment analysis (ssGSEA). Tumour immune dysfunction and exclusion (TIDE) was used to predict the efficacy of immunotherapy.

### 2.7 Drug sensitivity

The half maximal inhibitory concentration (IC50) values of paclitaxel, gefitinib, gemcitabine, and other anticancer drugs in LUAD samples were analysed. The pRRophetic package in R was used to calculate the IC50 of the drug (42).

### 2.8 Cluster analysis based on prognostic lncRNA

Potential molecular subgroups were explored using the ConsensusClusterPlus (CC) R package. Survival differences between clusters were analysed using the ‘survminer’ package. Subsequently, we used PCA and T-distributed stochastic neighbour embedding (t-SNE) to determine the discriminant degree of our cluster. TIMER (43), CIBERSORT (44, 45), QUANTISEQ (46), MCPCOUNTER (46), XCELL (45), and EPIC algorithm (47) were used to determine the immune infiltration of LUAD. In addition, we used ‘ggpubr’ and pRRophetic R packages to compare the differences in immune checkpoint-related gene expression and drug sensitivity between the three groups.

### 2.9 Cell culture

The Type Culture Centre of the Chinese Academy of Science (Shanghai, China) provided lung epithelial BEAS-2B cells and lung adenocarcinoma A549 and H1299 cell lines. BEAS-2B cells were cultured with DMEM medium (HyClone, USA). A549 and H1299 cells were cultured in RPMI-1640 medium (HyClone, USA). All media was supplemented with 10% FBS, 100 IU/mL penicillin G and 100 IU/mL streptomycin. The incubator conditions were set at 37°C and 5% CO_2_. The medium was changed every 2-3 days. Cells were passaged when the cell confluence reached 80-90%.

### 2.10 RNA extraction and real-time fluorescence quantitative PCR

Total RNA was isolated from cells using TRIzol reagent (Vazyme, China). We used HiScriptQRTSuperMix (Vazyme, China) reverse transcription RNA, cDNA as a template, and ChamQTMSYBRqPCRMaster (Vazyme, China) for qRT-PCR. We used GAPDH as an internal reference, and relative mRNA levels were calculated using the 2 ^-ΔΔCT^ method. All experiments were independently repeated three times. Primer sequences are shown in **Supplementary Table 2**.

### 2.11 Acquisition of immunohistochemical images

All the immunohistochemical (IHC) images used in this study were obtained from the Human Protein Atlas (HPA) database, with annotation conducted by certified pathologists (48). The staining intensity score was defined as follows: 0 for negative, 1 for weak, 2 for medium, and 3 for strong positive staining. The percentage of positive cells was defined as none for 0,< 25% cells for 1, 25-75% cells for 2, and< 75% cells for 3. The final IHC staining score = intensity score × percentage score.

### 2.12 Statistical analysis

The significance of the two groups of samples in the present study was tested using the Wilcox test, and the significance of the three groups and the above samples was tested using the Kruskal–Wallis test. The survival times of patients in the high- and low-risk groups were compared using Kaplan-Meier analysis, and the significance of the differences was evaluated using the log-rank test. Spearman’s correlation analysis was performed to determine the correlation of quantitative variables with non-normal distributions. Unless specified otherwise, all differences with p< 0.05 were considered statistically significant. PCR results were drawn using GraphPad Prism. Most analyses were performed using R software 4.1.1.

## 3 Results

### 3.1 Identification of HARlncRNAs in LUAD patients

**Figure 1:**
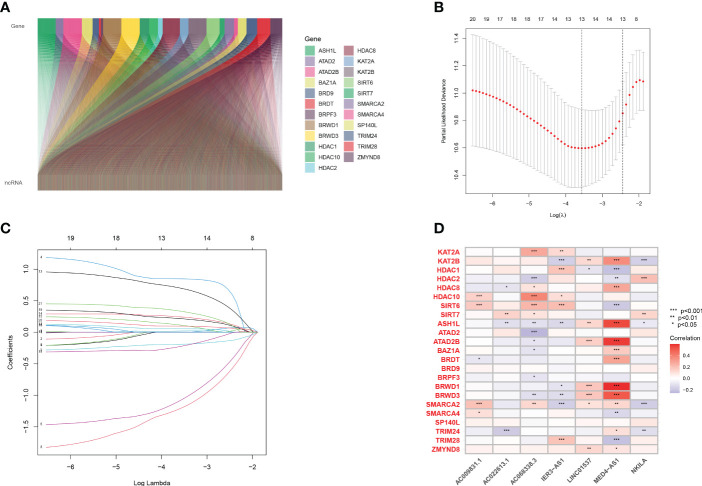
Screening prognostic lncRNA tags. **(A)** Co-expression analysis of 23 genes related to histone acetylation modification and lncRNAs in LUAD. **(B, C)** A 10-fold cross-validation of variable selection and LASSO coefficient distribution in LASSO regression analysis. **(D)** Correlation analysis of 7 lncRNAs and 23 genes related to histone acetylation modification.

First, co-expression analysis was used to find lncRNAs related to histone acetylation modification-related genes in LUAD, and the results were visualized in **Figure 1A**. A total of 21 lncRNAs strongly associated with overall survival (OS) (p< 0.05) were identified. Afterward, LASSO regression was performed, and 10-fold cross-validation was performed (**Figures 1B, C**). Multivariate Cox regression analysis was used to obtain seven lncRNAs for model construction (**Supplementary Table 3**). Afterward, we performed a correlation analysis between these seven most characteristic lncRNAs and histone acetylation modification-related genes through heat maps (**Figure 1D**). The expression of the seven HARlncRNAs in normal tissues and LUAD was shown in **Supplementary Figure 1**.

### 3.2 Construction of prediction features of LUAD HARlncRNAs

After multivariate Cox regression coefficients were obtained, a risk score formula was developed.


Risk score = LINC01537 × (1.08792810296881) + MED4−AS1× (−1.8004794989)+AC009831.1 × (−1.86593196441448) + IER3−AS1× (0.873358130447991) + AC022613.1 × (0.452982704837571) + AC068338.3× (−0.517189254806431) +NKILA × (0.352402305992526)

**Figure 2:**
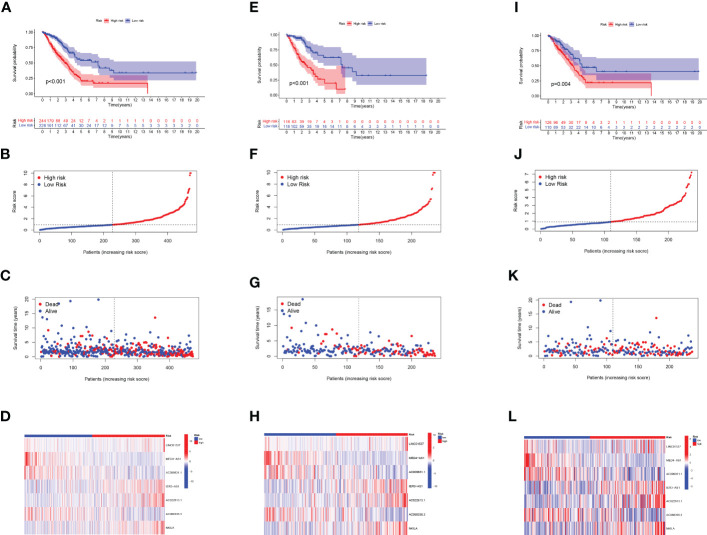
Evaluation of the label model of HARlncRNAs in the overall, training, and validation LUAD cohorts. **(A, E, I)** OS analysis of two subgroups in **(A)** total set, **(E)** trial set, and **(I)** validation set. **(B, F, J)** Risk score in **(B)** total set, **(F)** trial set, and **(J)** validation set. **(C, G, K)** Individual survival status in **(C)** total set, **(G)** trial set, and **(K)** validation set. **(D, H, L)** Heatmap of 7 lncRNAs expression in two subgroups in **(D)** total set, **(H)** trial set, and **(L)** validation set.

**Figure 3:**
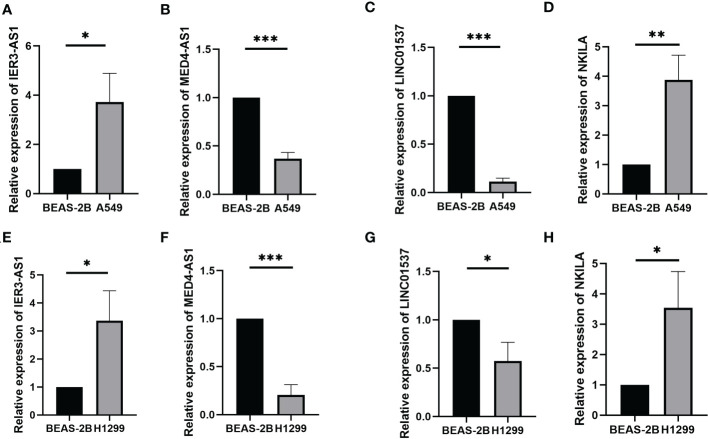
The expression of LINC01537, IER3-AS1, NKILA and MED4-AS1 in the A549 cell lines **(A–D)** and H1299 cell lines **(E–H)**. *p < 0.05; **p < 0.01; ***p < 0.001.

Prognostic risk models based on seven lncRNAs showed that OS was longer in the low-risk subgroup of the overall, training, and validation cohorts (**Figures 2A, E, I**). In addition, we visualised the distribution of risk scores and survival status and found that in all cohorts of LUAD patients, higher risk scores corresponded with more death events (**Figures 2B, C, F, G, J, K**). Simultaneously, using the expression heatmap, we confirmed that LUAD patients with high expression of LINC01537, IER3-AS1, AC022613.1, and NKILA were associated with high-risk scores. In contrast, AC068338.3, AC009831.1, and MED4-AS1 were highly expressed in patients with a low-risk score (**Figures 2D, H, L**). In addition, we analysed the expression of LINC01537, IER3-AS1, NKILA, and MED4-AS1 in cell lines (**Figures 3A–H**). These results are consistent with those of the public database.

### 3.3 Accuracy of models in clinical application

**Figure 4:**
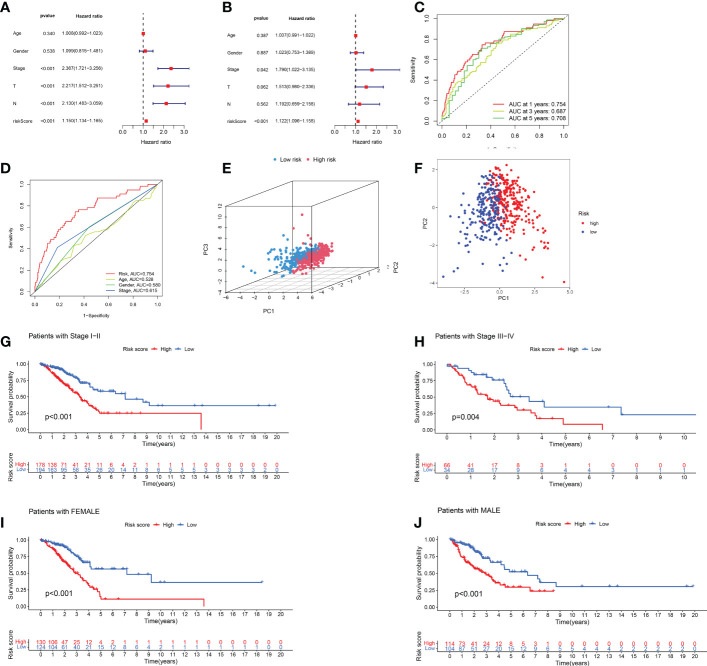
Validation of prognostic models for lncRNAs associated with histone acetylation modification. **(A, B)** A multivariate and univariate analysis of clinicopathological factors associated with OS in patients with LUAD. **(C)** One-, three-, and five-year OS prediction models **(D)** Risk scores and other clinical factors are compared using ROC curves. **(E, F)** PCA analysis of risk model based on expression profiles of 7 HARlncRNAs. **(G, H)** Stratified survival analysis based on clinical stages of LUAD. **(I, J)** Stratified survival analysis based on patient gender.

We evaluated the prognostic ability of the independent prognostic models using univariate and multivariate Cox regression analyses. Both analyses showed that risk score was an independent prognostic factor (**Figures 4A, B**). In addition, we found that the AUCs were more prominent at 1, 3, and 5 years, indicating that the constructed model predicted patients with high accuracy (**Figure 4C**). The constructed model was further compared with the other clinical characteristics of ROC curves. The risk scores had the highest AUC values among these factors (**Figure 4D**). We further verified the prognostic value of the model using PCA (**Figures 4E, F**). The risk model could effectively distinguish between patients. The C-index of the risk score was the highest (**Supplementary Figure 2**). These results confirmed that the risk model based on the expression profiles of the seven HARlncRNAs might be a potential prognostic marker.

Afterward, we performed a stratified analysis of staging and sex. Low-risk patients showed consistently better overall survival across early- and advanced-stage, female, and male patients (**Figures 4G–J**).

### 3.4 Nomogram for predicting patient prognosis

**Figure 5:**
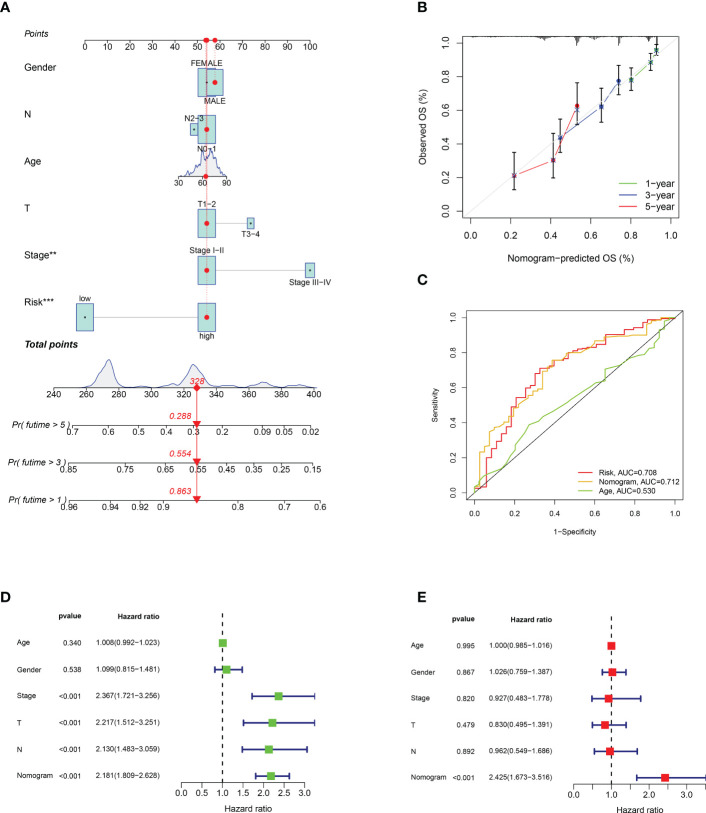
Establishment and verification of nomogram. **(A)** Nomogram with risk score model and clinicopathological features. **(B)** Calibration diagram of the nomogram. **(C)** TCGA-LUAD risk score and clinical characteristics ROC curve. **(D, E)** Univariate and multivariate Cox regression analyses of the nomogram. **p < 0.001; ***p < 0.001.

We developed an OS nomogram including sex, lymph node metastasis, tumour size, age, stage, and calculated risk score to estimate the survival probability of patients. The results showed that when the score was 328, the 1-, 3-, and 5-year survival rates were 0.863, 0.554, and 0.288, respectively (**Figure 5A**). **Figure 5B** shows the calibration curves for LUAD at 1, 3, and 5 years, indicating that the nomogram could reliably predict OS in these patients. In addition, the ROC curve showed that the nomogram had a stronger predictive value than age and the prognostic risk score model (**Figure 5C**). In univariate Cox regression analysis, stage, lymph node metastasis, tumour size, and nomogram were independent prognostic factors (**Figure 5D**). In multivariate regression analysis, the nomogram remained the only independent prognostic factor (**Figure 5E**).

### 3.5 Differences in the biological pathways of differential genes

**Figure 6:**
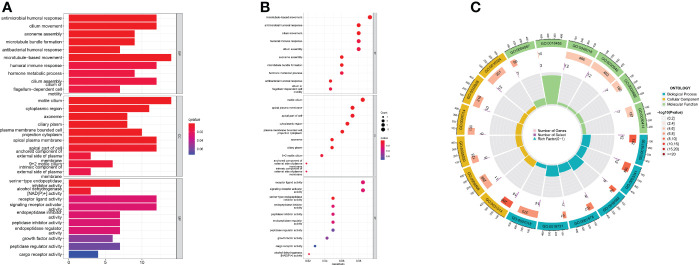
Focusing on the functional pathways of DEGs. **(A)** Bar chart **(B)** Bubble diagram **(C)** Circle diagram of GO enrichment analysis.

We found differentially expressed genes between the two groups using differential analysis (**Supplementary Table 4**). GO enrichment results showed that the differentially expressed genes were enriched in the processes of microtubule-based movement, antimicrobial humoral response, and cilium movement in BP, whereas the CC process focused on the processes of the motile cilium, apical plasma membrane, and apical part of the cell, and MF was enriched in receptor-ligand activity and signal receptor activator activity. (**Figures 6A–C**). The GO digital index numbers are listed in **Supplementary Table 5**.

### 3.6 Somatic cell variants and risk score models

**Figure 7:**
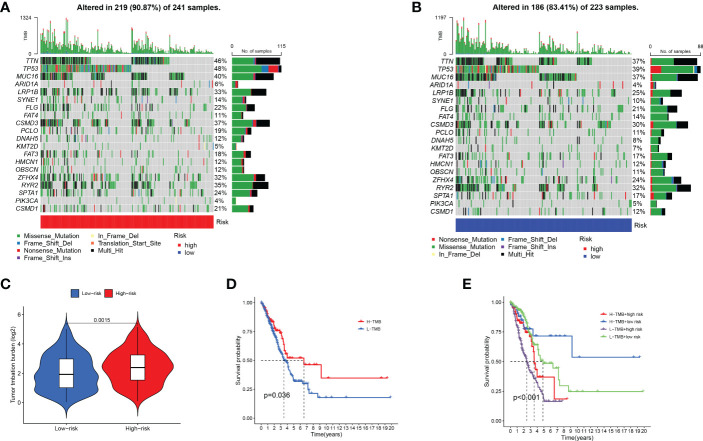
Impact of risk score models on somatic gene alterations and TMB. **(A, B)** Comparison of gene mutation rates between two subgroups. **(C)** Differential analysis of TMB between two subgroups. **(D)** The prognostic role of TMB in LUAD patients. **(E)** The prognostic role of combined risk score model and TMB in LUAD patients.

Through analysis of the waterfall diagram, we found that TP53, TTN, and MUC16 ranked in the top three in the two groups (**Figures 7A, B**). Afterward, we downloaded and analysed immunohistochemical images of TP53, TTN, and MUC16 in normal and LUAD clinical samples (**Supplementary Figure 3–5**). The results showed that the staining scores of TP53 and MUC16 in tumour tissues were higher, whereas the TTN staining fraction between both tumour and alveolar tissues was not significant. (**Supplementary Table 6**). **Figure 7C** shows that there were differences in TMB expression between the two groups. In addition, survival analysis showed better prognosis in patients with high TMB (**Figure 7D**). A combination of the model and TMB was analysed, and the results showed that patients with low risk and high TMB had a better prognosis (**Figure 7E**). The results showed our constructed prediction model was not affected by TMB status, indicating that our model was more accurate than TMB in predicting prognosis.

### 3.7 Analysis of immune microenvironment and immunotherapy

**Figure 8:**
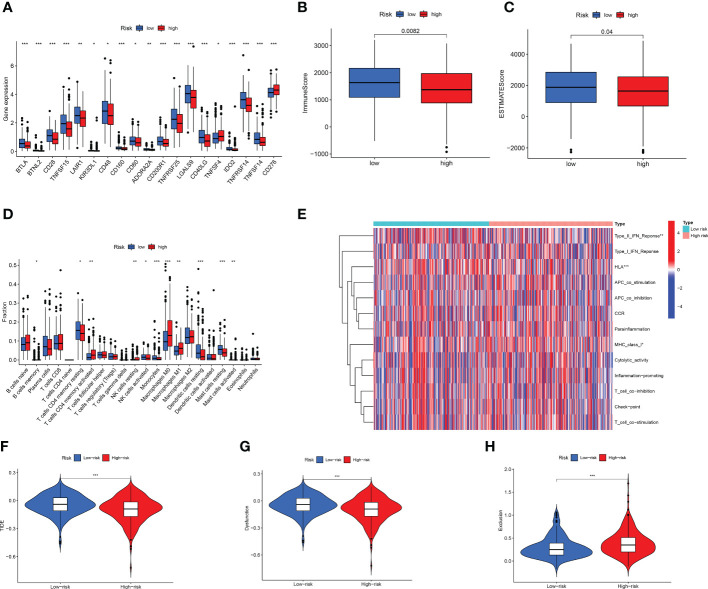
Cell infiltration in LUAD tumor microenvironment. **(A)** High-risk compared to low-risk immune checkpoint gene expression. **(B, C)** Differences in **(B)** ImmuneScore and **(C)** StromalScore between two groups. **(D)** Evaluation of immune cell infiltration. **(E)** Analysis of immune-related functions. **(F–H)** TME was evaluated based on TIDE for two subgroups. *p < 0.05; **p < 0.01; ***p < 0.001.

At present, there is an increasing number of ICIs for tumours. We analysed the immunohistochemical staining of PD-L1 (**Supplementary Figure 6**) and PD-L2 (**Supplementary Figure 7**) in patients with LUAD. The results of the immunohistochemical scoring are shown in **Supplementary Table 6**. Thereafter, we analysed the differences in ICI gene expression between the two groups. We found that most genes were highly expressed in the low-risk group, including CD28, LAIR1, KIR3DL1, CD48, CD80, and ADORA2 (**Figure 8A**). Based on the ESTIMATE algorithm, we analysed the abundance of immune cells between the two groups. We found that the low-risk group had higher immune and estimated scores, whereas there was no difference in stromal scores (**Figures 8B, C**; **Supplementary Figure 8**).

Afterward, we used CIBERSORT to analyse 22 infiltrating immune cells. The two groups exhibited different immunologic profiles. M1 macrophages and CD8+ T cells were significantly elevated in the high-risk group, whereas resting dendritic and mast cells were significantly enriched in the low-risk group (**Figure 8D**). Next, we used ssGSEA to analyse immune-related functions. We found that the type II IFN response and HLA immune-related functions were more active in the low-risk group than in the high-risk group (**Figure 8E**). We used multiple algorithms to analyse the immune infiltration of high- and low-risk LUAD patients, which can be considered to be an external verification (**Supplementary Figures 9–16**). TIDE can predict whether patients will benefit from the use of ICIs, and the higher the score, the more prone patients were to immune escape (49). **Figures 8F–H** shows that the TIDE score of the low-risk group was higher, suggesting that patients in the low-risk group were more prone to immune escape. Similar results were obtained for the TIDE score of the test group (**Supplementary Figure 17**). T cell dysfunction was more significant in the low-risk group. However, immune exclusion was observed more frequently in the high-risk group.

### 3.8 Prediction of drug sensitivity by the risk model

**Figure 9:**
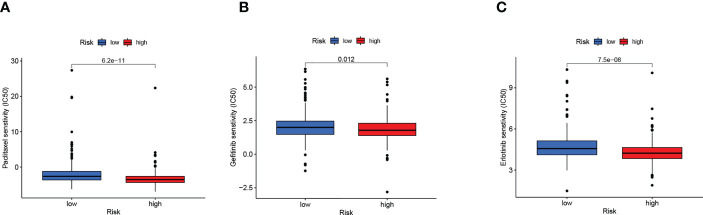
Risk score guide clinical. **(A–C)** Differences in sensitivity to clinical drugs between two subgroups.

Sensitivity analysis of the three common therapeutic drugs for LUAD showed that high-risk patients might have a higher sensitivity to paclitaxel, gefitinib, and erlotinib (**Figures 9A–C**), suggesting that the risk score model might help to identify LUAD patients that are more likely to benefit from chemotherapy and targeted therapy.

### 3.9 Identifying hot and cold tumours and ICIs sensitivity

**Figure 10:**
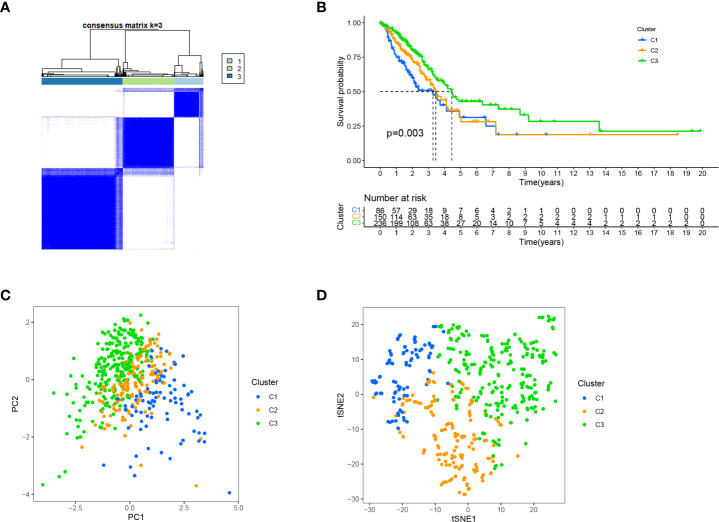
Distinguishing different clusters. **(A)** Patients are classified into three categories by ConsensusClusterPlus. **(B)** Kaplan-Meier survival curve in the three clusters. **(C, D)** PCA and t-SNE of three cluster.

Different immune microenvironments have different subtypes, resulting in different immunotherapy effects (50, 51). Clusters can distinguish between hot and cold tumours and guide immunotherapy (52–55). Therefore, we used the R package ‘ConsensusClusterPlus’ to group the patients into three clusters based on cluster analysis. (**Figure 10A**; **Supplementary Figure 18**). Survival analysis showed that the OS of cluster 3 was the longest (**Figure 10B**). T-SNE and PCA revealed that these three clusters were distinguishable (**Figures 10C, D**).

**Figure 11:**
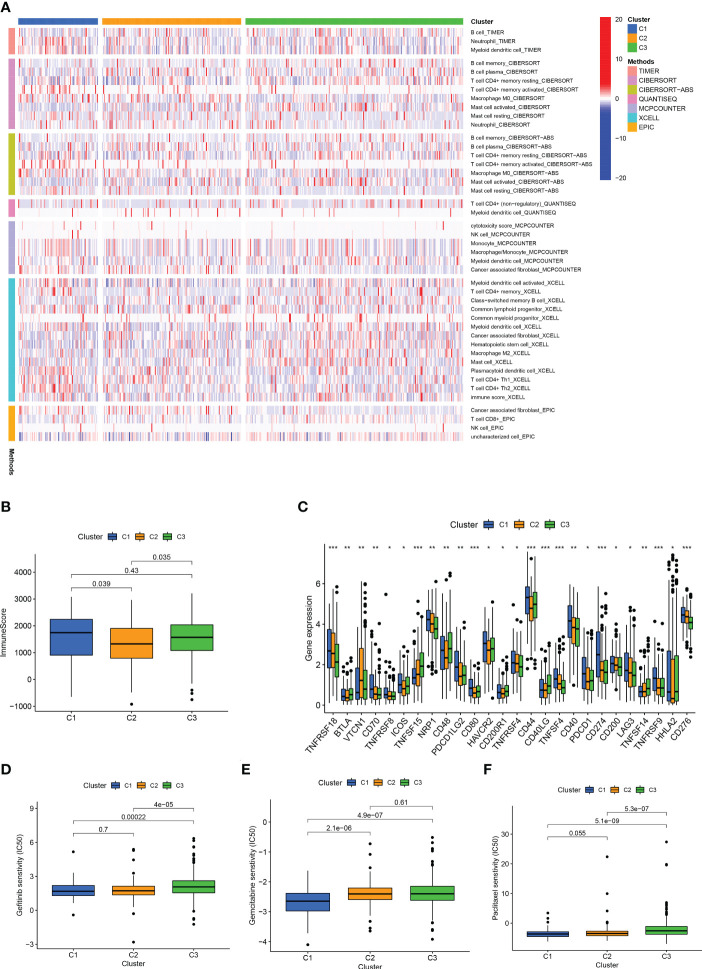
Different cluster immunity and drug sensitivity. **(A)** Heat maps of immune cells in different clusters. **(B)** Immune scores of different clusters. **(C)** Differential expression of immune checkpoint genes in different clusters. **(D–F)** IC50 of different clusters in gefitinib, gemcitabine, paclitaxel. *p < 0.05; **p < 0.01; ***p < 0.001.

Afterward, we analysed the infiltration of immune cells on different platforms, and the results showed that clusters 1 and 3 had a higher degree of CD8+ T immune cell infiltration (**Figure 11A**). In addition, clusters 1 and 3 had higher immune scores than cluster 2 (**Figure 11B**). Almost all ICIs, including LAG3 and CD274, were highest in cluster 1 (**Figure 11C**). Increased infiltration of CD8+ T immune cells, activation of immune checkpoints, such as CD274 and LAG3, and high immune scores play a crucial role in hot tumours (56, 57). Therefore, we classified clusters 1 and 3 as hot tumours that were more sensitive to immunotherapy (56). We then analysed the sensitivity of different clusters to drugs. The results showed that clusters 1 and 2 might be more sensitive to gemcitabine, paclitaxel, and gefitinib (**Figures 11D–F**). Based on the above analysis results, we can improve research on immunotherapy in LUAD patients and improve the accuracy of patient treatment.

## 4 Discussion

The present study constructed a risk-scoring model based on seven HARlncRNAs related to prognosis. ROC curve, univariate analysis, and multivariate analysis were used to verify the accuracy of the model. Patients in the high-risk group showed higher immune cell infiltration and were predicted to be more sensitive to chemotherapy, targeted therapy, and immunotherapy drugs. Therefore, the risk-scoring model based on seven prognostic lncRNAs might correctly estimate the prognosis of LUAD patients and identify potential candidates for chemotherapy, targeted therapy, and immunotherapy. Although our risk model can predict patient prognosis and guide drug treatment, it cannot identify hot and cold tumours. Clusters can distinguish between hot and cold tumours and guide immunotherapy (52–55). Therefore, we divided the patients into three clusters. Clusters 1 and 3 exhibited higher CD8+ T immune cell infiltration. CD8 + T cells are the main driver of antitumour immunity (57). In addition, clusters 1 and 3 had higher immune scores and higher CD274 and LAG3 than cluster 2. Therefore, we suggested that clusters 1 and 3 can be considered hot tumours (56). Furthermore, clusters 1 and 3, as hot tumours, might be more sensitive to immunotherapy (51, 56). This suggested that our classification could not only predict patient prognosis, but also guide individualised treatment. More importantly, based on these lncRNAs as liquid biopsies, it is more convenient and effective to distinguish between hot and cold tumours (58).

Lung cancer has the highest mortality rate among all malignancies (59). Despite significant advances over the past few decades in early screening and treatment, the prognosis of patients with LUAD remains dismal. The molecular pathogenesis of lung cancer involves mutations and disorders of oncogenes and tumour suppressor genes (60). Additionally, epigenetic abnormalities play key roles in the development and progression of lung cancer (61, 62). Therefore, an in-depth study of epigenetics will help to identify new disease biomarkers.

In our model, we used seven different HARlncRNAs, namely LINC01537, MED4-AS1, AC009831.1, IER3-AS1, AC022613.1, AC068338.3, and NKILA. INC01537 was reported to be a tumour suppressor located on the human chromosome 11q13.4 (63). Gong et al. found that LINC01537 was expressed at low levels in lung cancer and that phosphodiesterase 2A (PDE2A) was the target of LINC01537. LICN01537 not only inhibited tumour growth and metastasis, but also increased sensitivity to nilotinib (64). Wu et al. found that MED4-AS1 was downregulated in LUAD; however, its role is not fully understood (65). Mahale et al. demonstrated that the carcinogenic properties of IER3 and IER3-AS1 are determined by their interaction with HnRNPK (66). AC022613.1 was highly expressed in LUAD and associated with a poor prognosis. In addition, AC022613.1 affects the prognosis of many other cancers (67). Lu et al. found that the expression of AC068338.3 was lower in LUAD tissues and cell lines than in normal tissues (68). NF-kappa B-interacting lncRNA (NKILA) is upregulated by NF-κB in breast cancer (69, 70). Lu et al. found that the expression of NKILA was downregulated in NSCLC tissues (71). To date, no relevant studies have been conducted on AC009831.1. Our study shows that LINC01537, IER3 − AS1, AC022613.1, and NKILA are high-risk lncRNAs, and MED4 − AS1, AC009831.1, and AC068338.3 are low-risk lncRNAs.

Immune checkpoint inhibition alone for PD-L1 high-expressing tumours and combined with cytotoxic chemotherapy for PD-L1 low-expressing tumours have become the standard of care for first-line treatment of advanced NSCLC; however, this treatment approach does not have a high overall effect. More importantly, many patients develop primary or secondary resistance to immunotherapy (72, 73). TMB was originally proposed as an indicator of the number of neoantigens produced by mutated genes in tumour cells. High TMB is thought to lead to increased tumour neoantigen expression that would be cross-presented and activate tumour-specific immune responses (74). Alternatively, TMB could be predictive of immunotherapy efficacy. PD-L1 expression level and TMB status were predictive biomarkers of anti-PD-L1 activity in the Checkmate 026 trial (75). Furthermore, the KEYNOTE-158 study showed that patients with high TMB were more likely to benefit from immunotherapy (76). Therefore, TMB has been accepted as a predictor of immunotherapy in advanced NSCLC and was recommended by the recent NCCN guidelines (77). Given the good predictive value of TMB, we explored the relationship between TMB and the risk-score model. To further test the ability of the established model to predict patients ‘ response to immunotherapy, we analysed the TIDE scores of the two groups, and found that the high-risk group had a lower TIDE score, indicating that the high-risk group may respond better to immunotherapy. These results suggest that our established model could predict the effectiveness of immunotherapy in patients with LUAD.

Chemotherapy is one of the most important systemic treatments for patients with advanced NSCLC. Combined paclitaxel and platinum, as a first-line treatment for stage IV NSCLC without driver gene mutations, improves patient survival (78). Targeted therapy is the basis for the treatment of advanced NSCLC harbouring driver gene mutations. Studies comparing gefitinib with chemotherapy in patients with EGFR mutations have shown that gefitinib significantly improved PFS (79). Additionally, the CSCO guidelines (version 2020) recommend erlotinib as the first-line treatment for stage IV NSCLC with EGFR mutations (80). The results of our study suggest that the high-risk group may be more sensitive to the chemotherapeutic drug paclitaxel and targeted therapy drugs, including gefitinib and erlotinib. In conclusion, we developed a scoring model that can provide a reference for drug selection in patients with LUAD.

Although our risk model has good predictive potential, this study had several limitations. First, regardless of all the information we searched for in the GSE31210, GSE50081, and GSE72094 series from GEO, we could not obtain sufficient information for lncRNA; therefore, we did not use it as an external validation queue. Liu et al. suspected that there were deviations and limitations between commercial microarray data and TCGA data (53). However, the data of our immune cell bubble map comes from multiple platforms and can be used for the verification of external data (55). Second, in the future, a large number of clinical samples should be collected to confirm the practical application value of our model.

## Conclusion

This study provides detailed evidence on the substantial interaction between lncRNAs of histone acetylation modification-related genes and the prediction of LUAD prognosis. Risk scores were identified as potential prognostic markers for LUAD, and the practical applicability of the model was investigated in terms of its sensitivity to chemotherapy, targeted drugs, and immunotherapy.

## Data availability statement

The original contributions presented in the study are included in the article/**Supplementary Material**. Further inquiries can be directed to the corresponding authors.

## Author contributions

RL, TQ, QZ, QW, and CX designed this study. RL, TQ, and QZ collected the data. RL, FH, CJ, and ZL analyzed the data. RL, TQ, QZ, FH, XZ, ZL and CJ wrote the main manuscript text. FH did the experiment. CX and QW revised the manuscript. All authors reviewed the manuscript. All authors contributed to the article and approved the submitted version.
