# Supplementary figures and images for: Histone acetylation-related IncRNA: Potential biomarkers for predicting prognosis and immune response in lung adenocarcinoma, and distinguishing hot and cold tumours
Source: Front Immunol. 2023 Mar 17;14:1139599. doi: 10.3389/fimmu.2023.1139599 (PMC10064094; doi:10.3389/fimmu.2023.1139599)

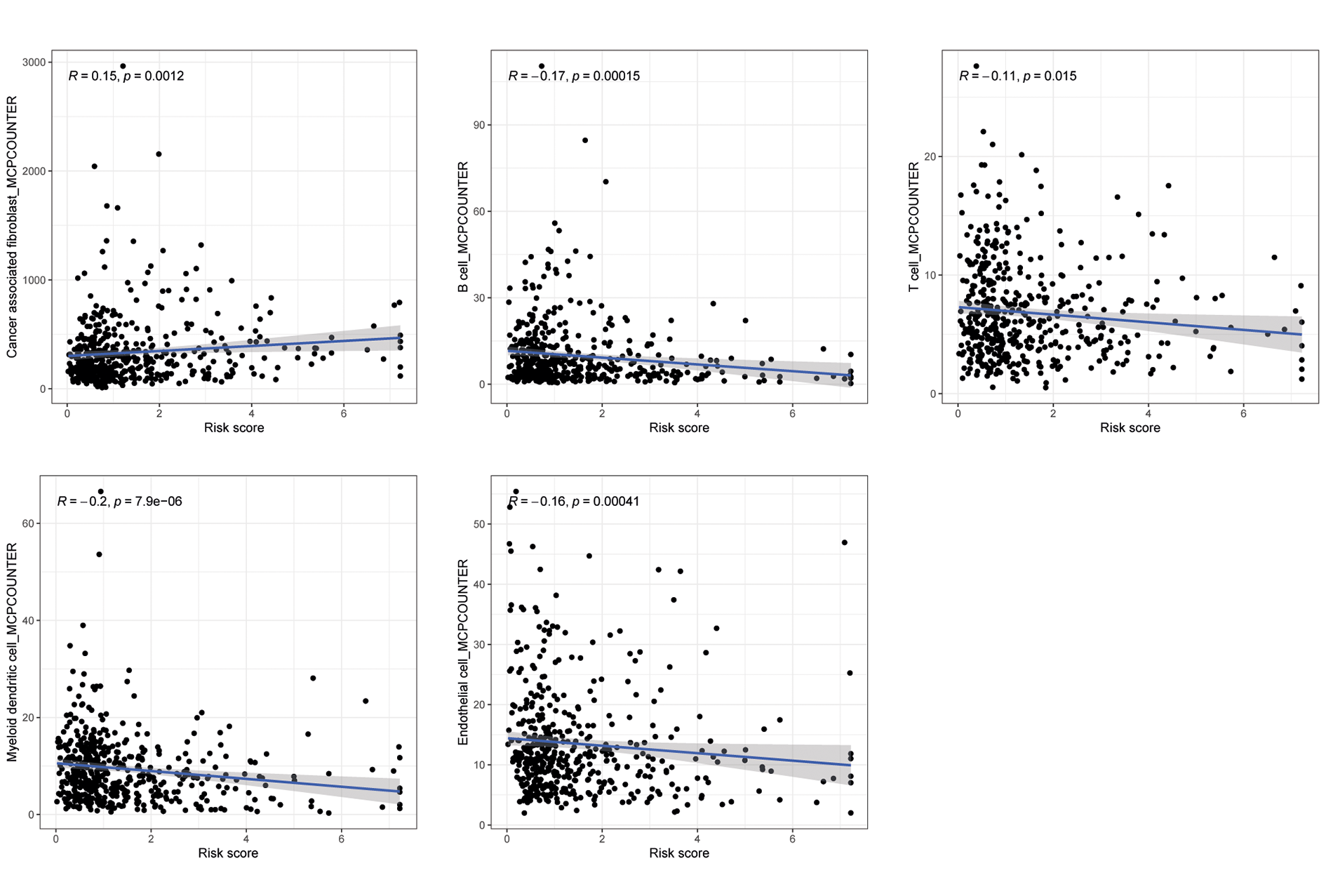

Supplement: Supplementary file 1 [file DataSheet_1.zip › Image 12.TIF]

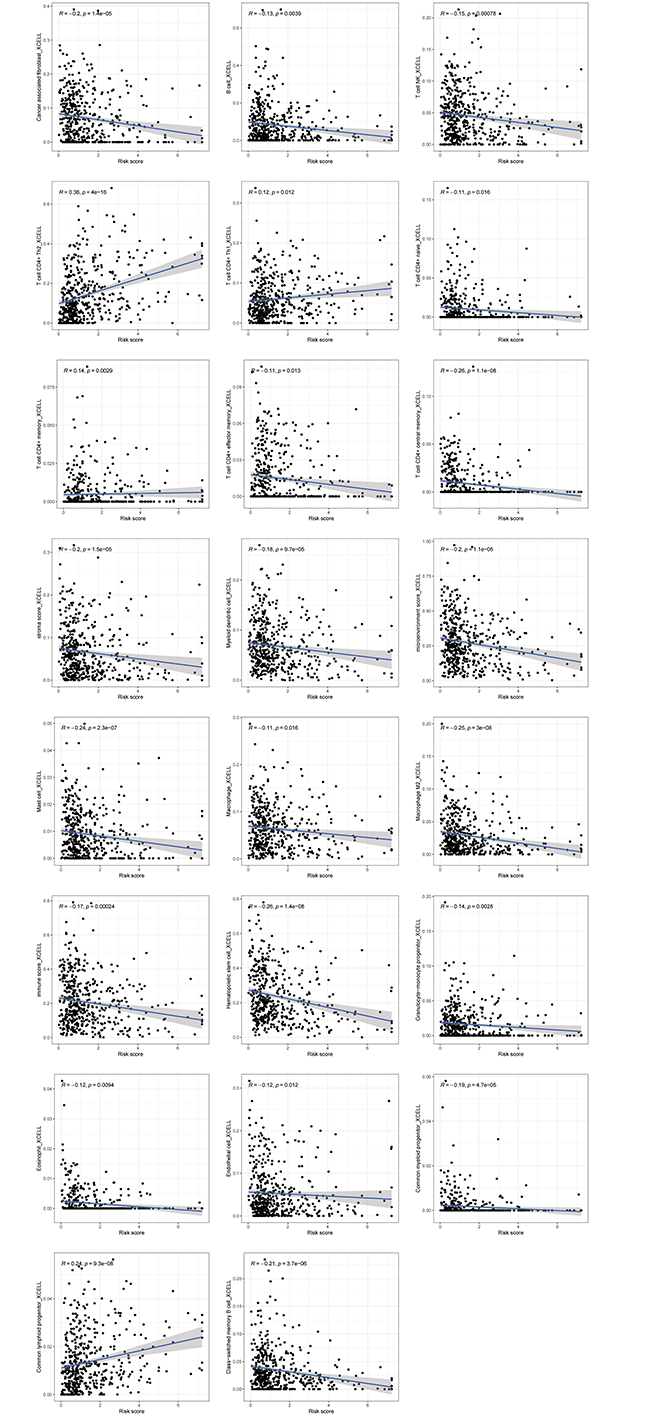

Supplement: Supplementary file 1 [file DataSheet_1.zip › Image 13.TIF]

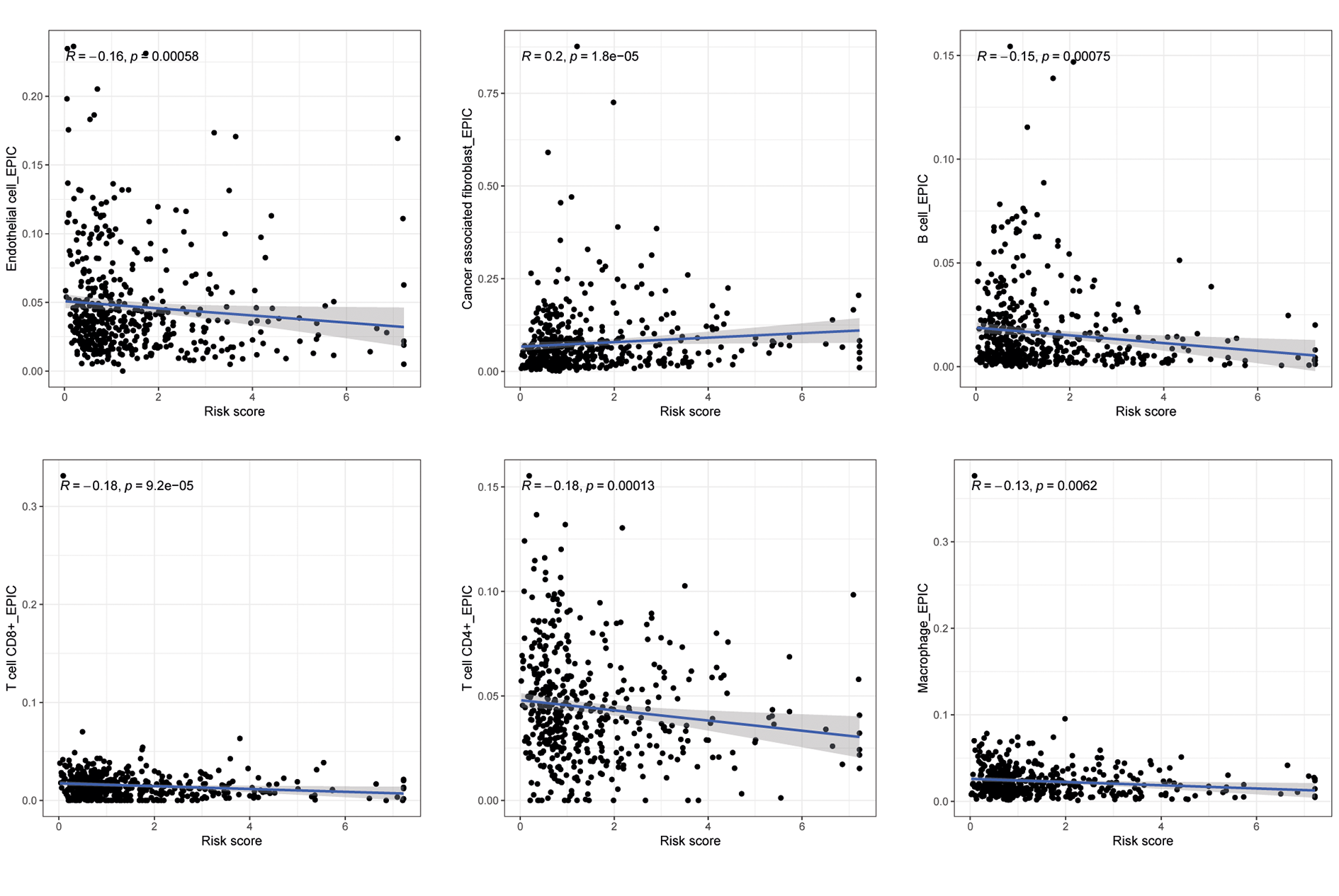

Supplement: Supplementary file 1 [file DataSheet_1.zip › Image 14.TIF]

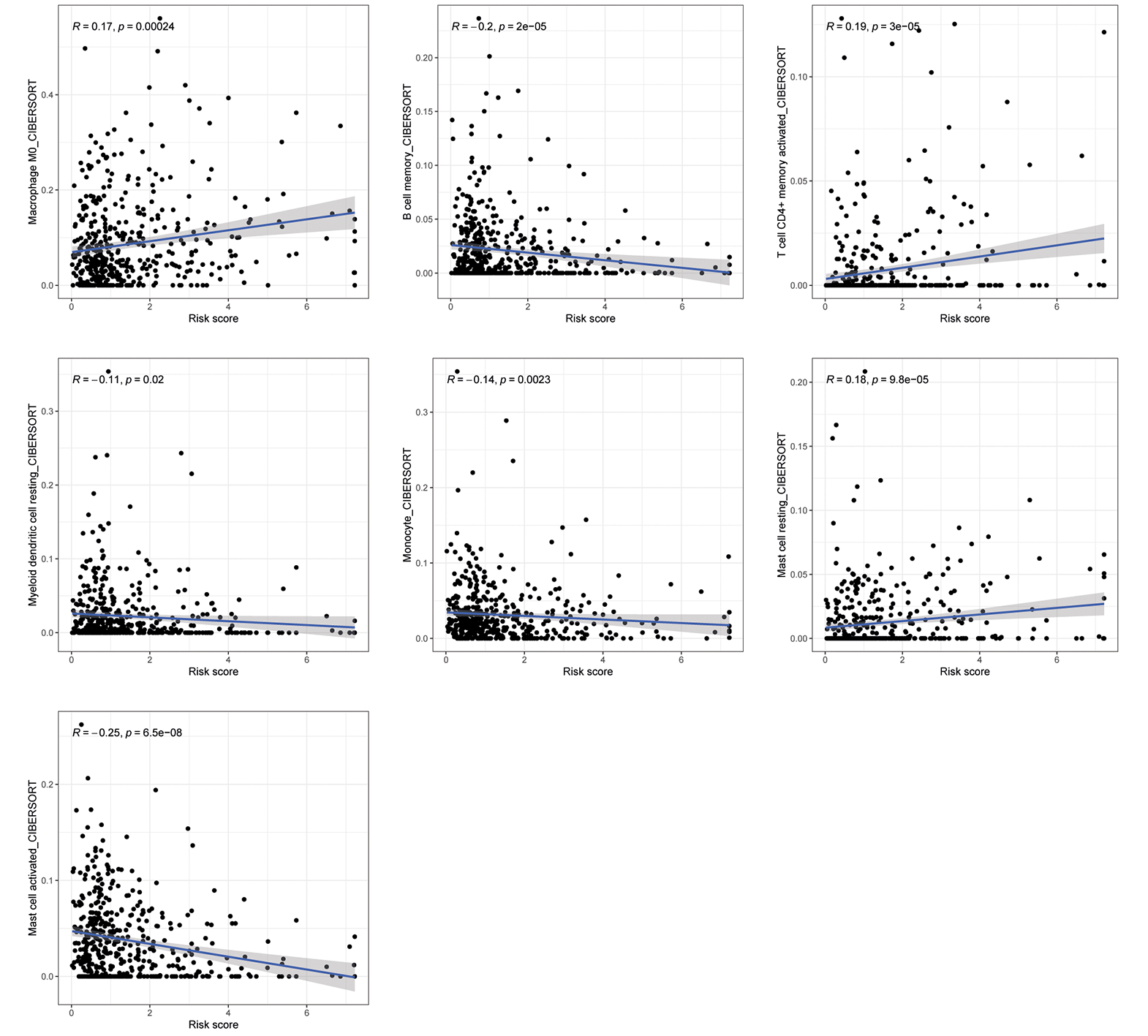

Supplement: Supplementary file 1 [file DataSheet_1.zip › Image 15.tif]

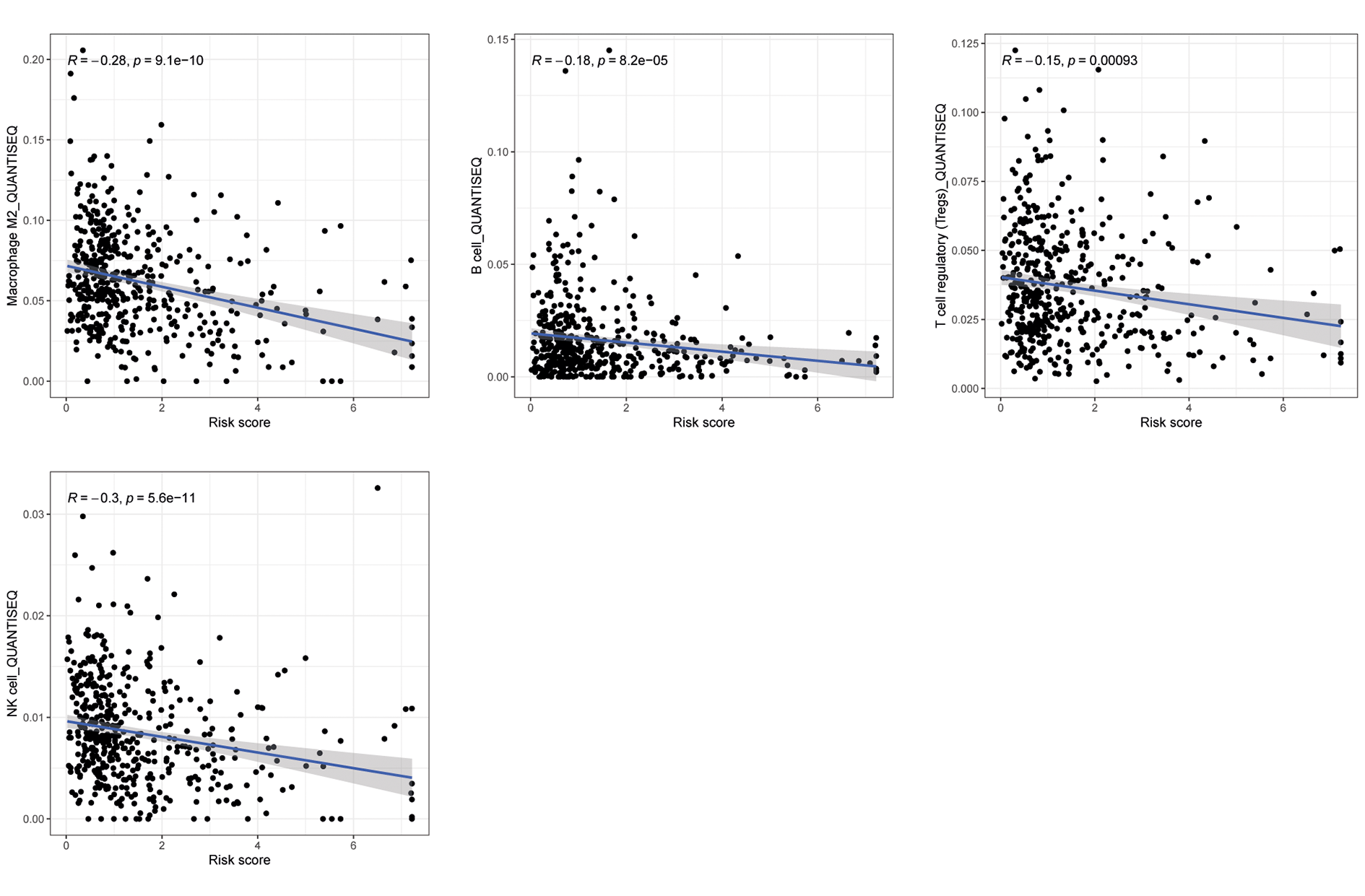

Supplement: Supplementary file 1 [file DataSheet_1.zip › Image 16.tif]

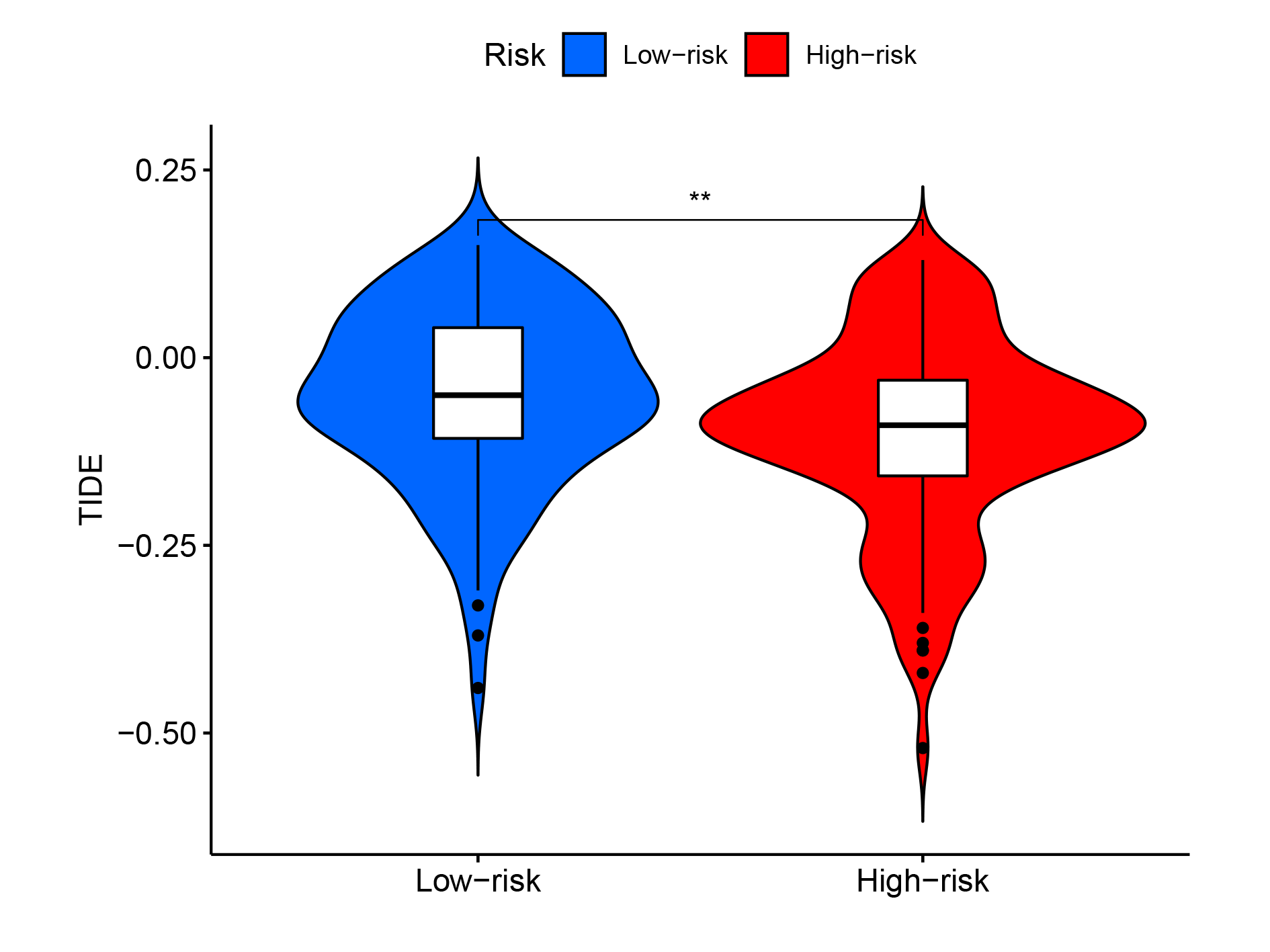

Supplement: Supplementary file 1 [file DataSheet_1.zip › Image 17.tif]

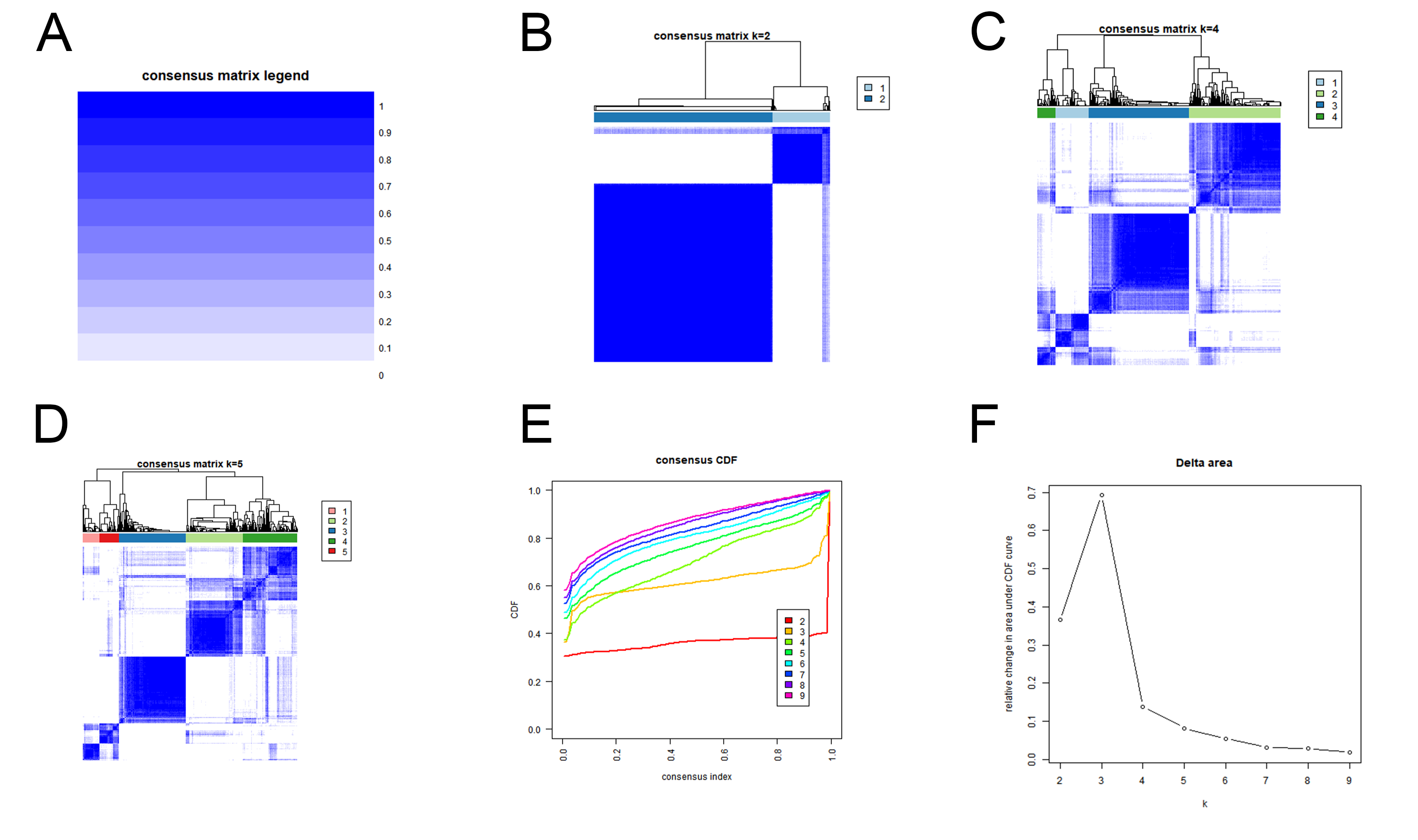

Supplement: Supplementary file 1 [file DataSheet_1.zip › Image 18.TIF]

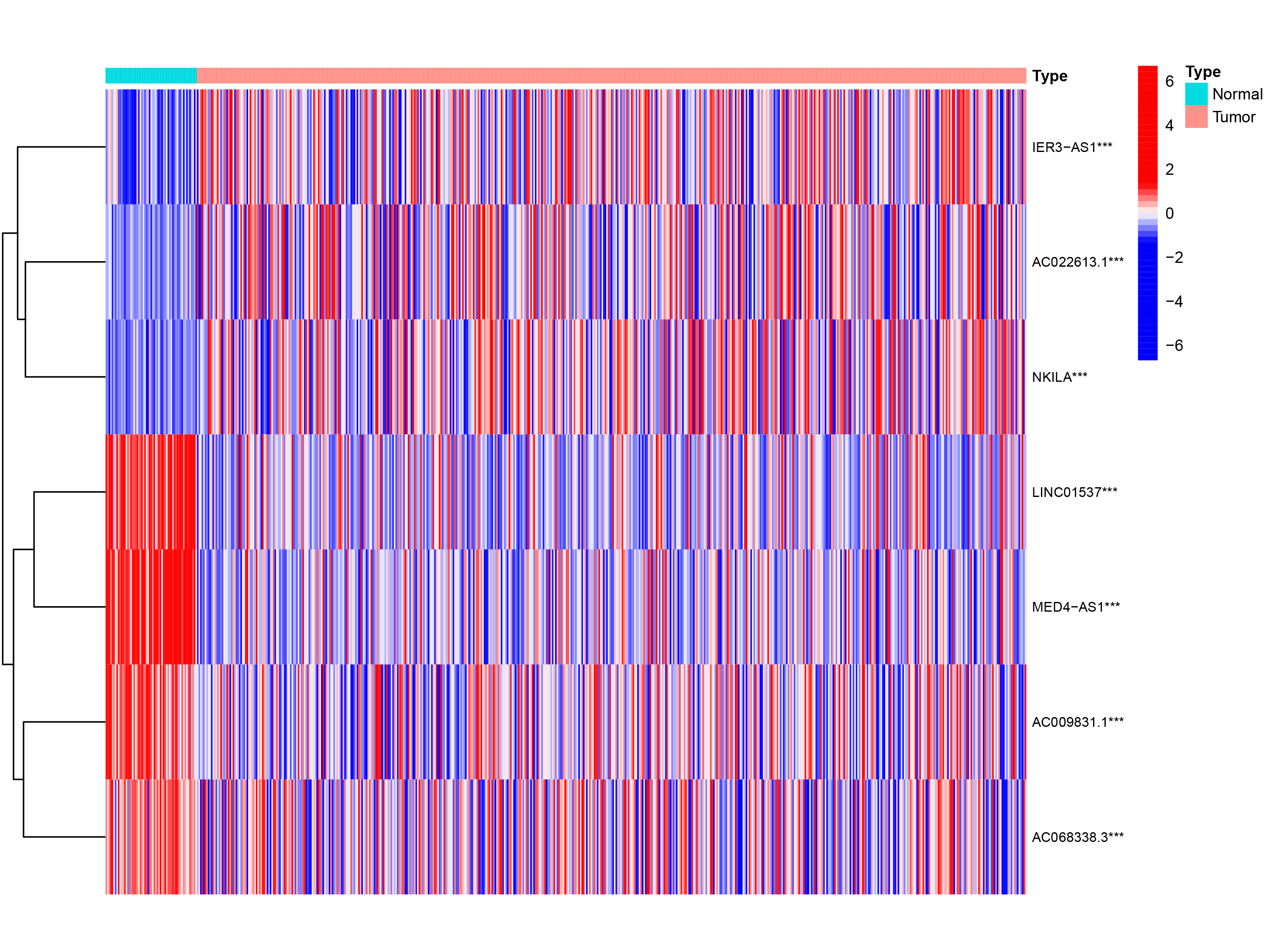

Supplement: Supplementary file 1 [file DataSheet_1.zip › Image 1.TIF]

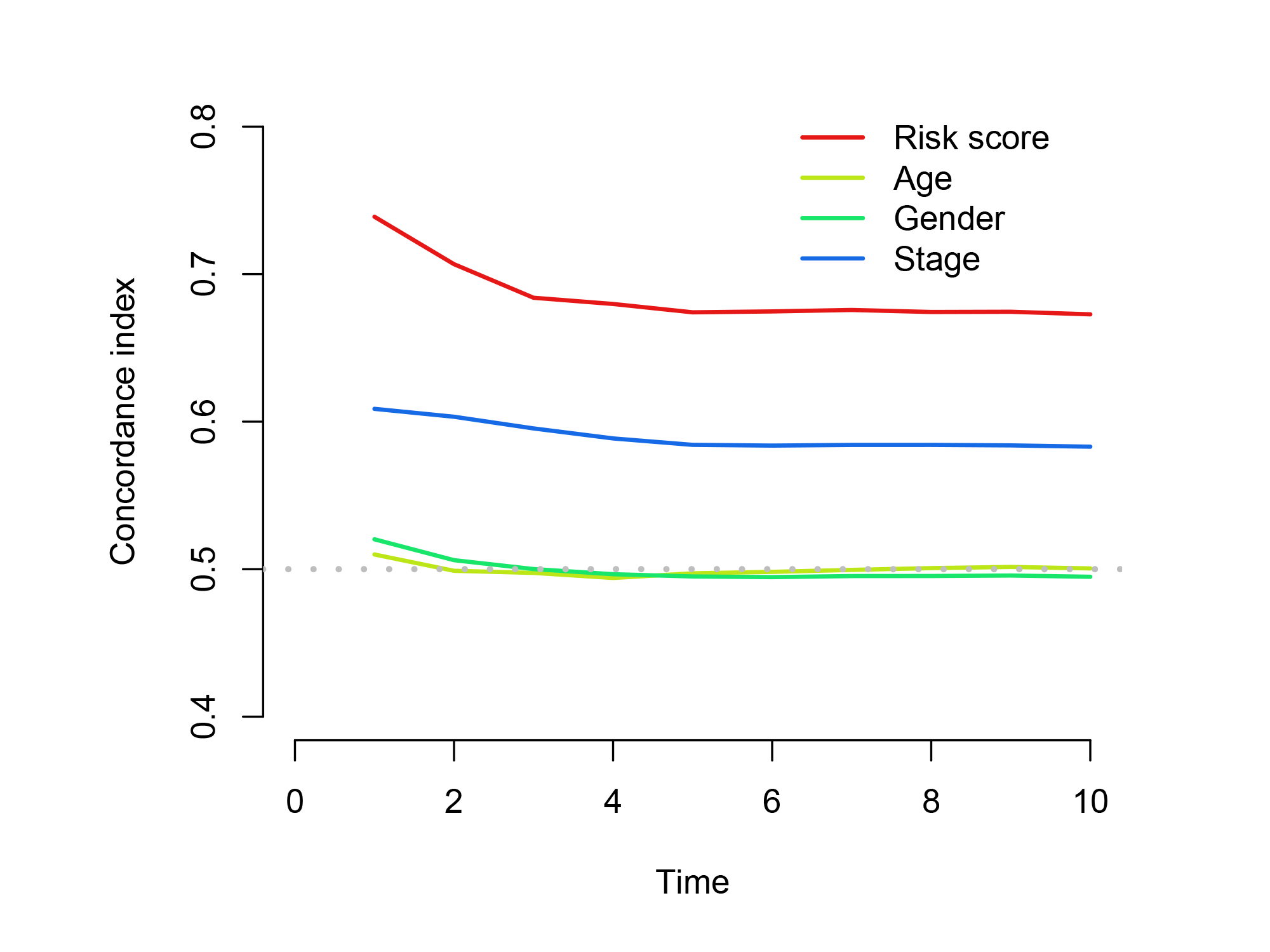

Supplement: Supplementary file 1 [file DataSheet_1.zip › Image 2.TIF]

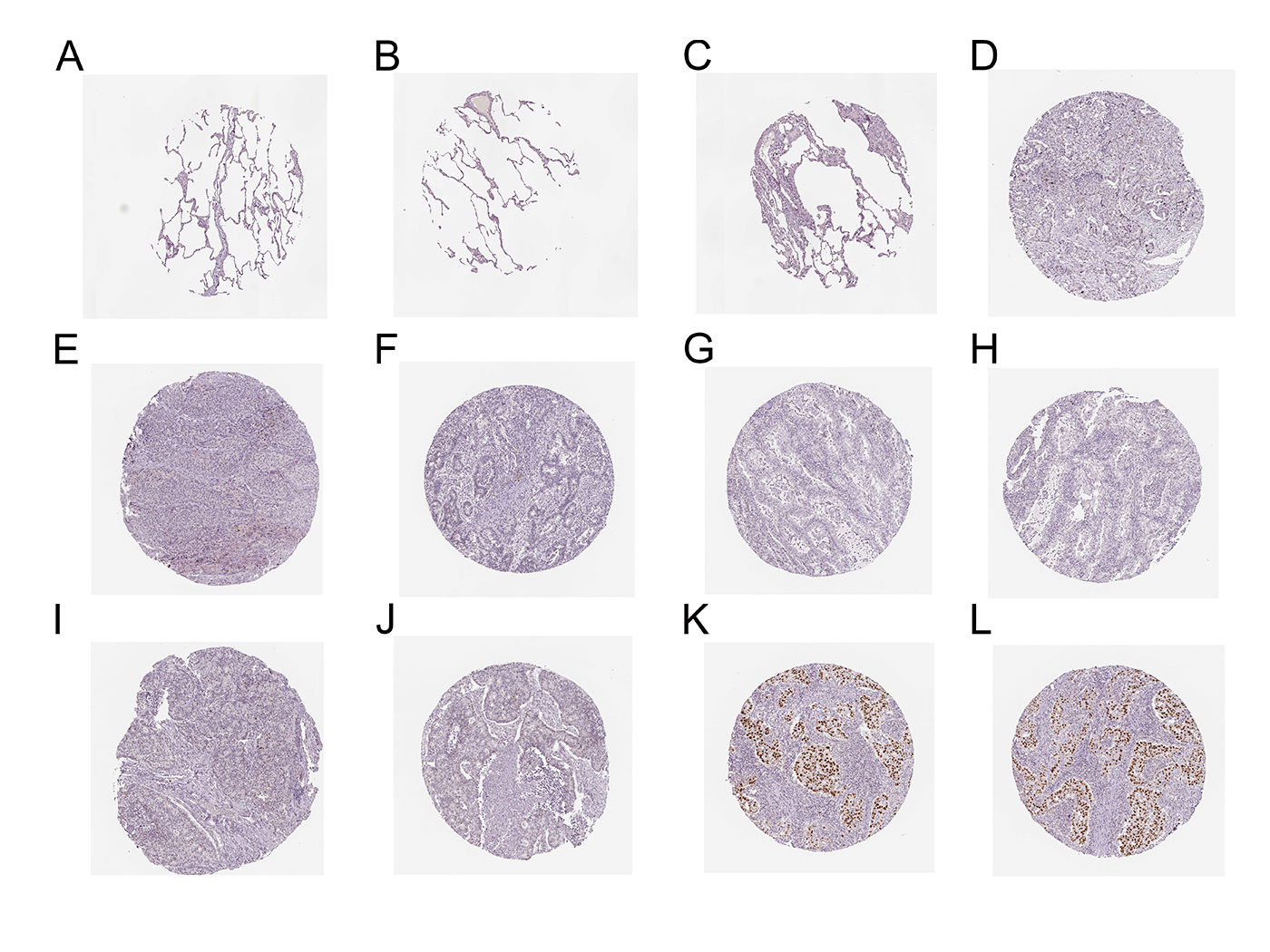

Supplement: Supplementary file 1 [file DataSheet_1.zip › Image 3.TIF]

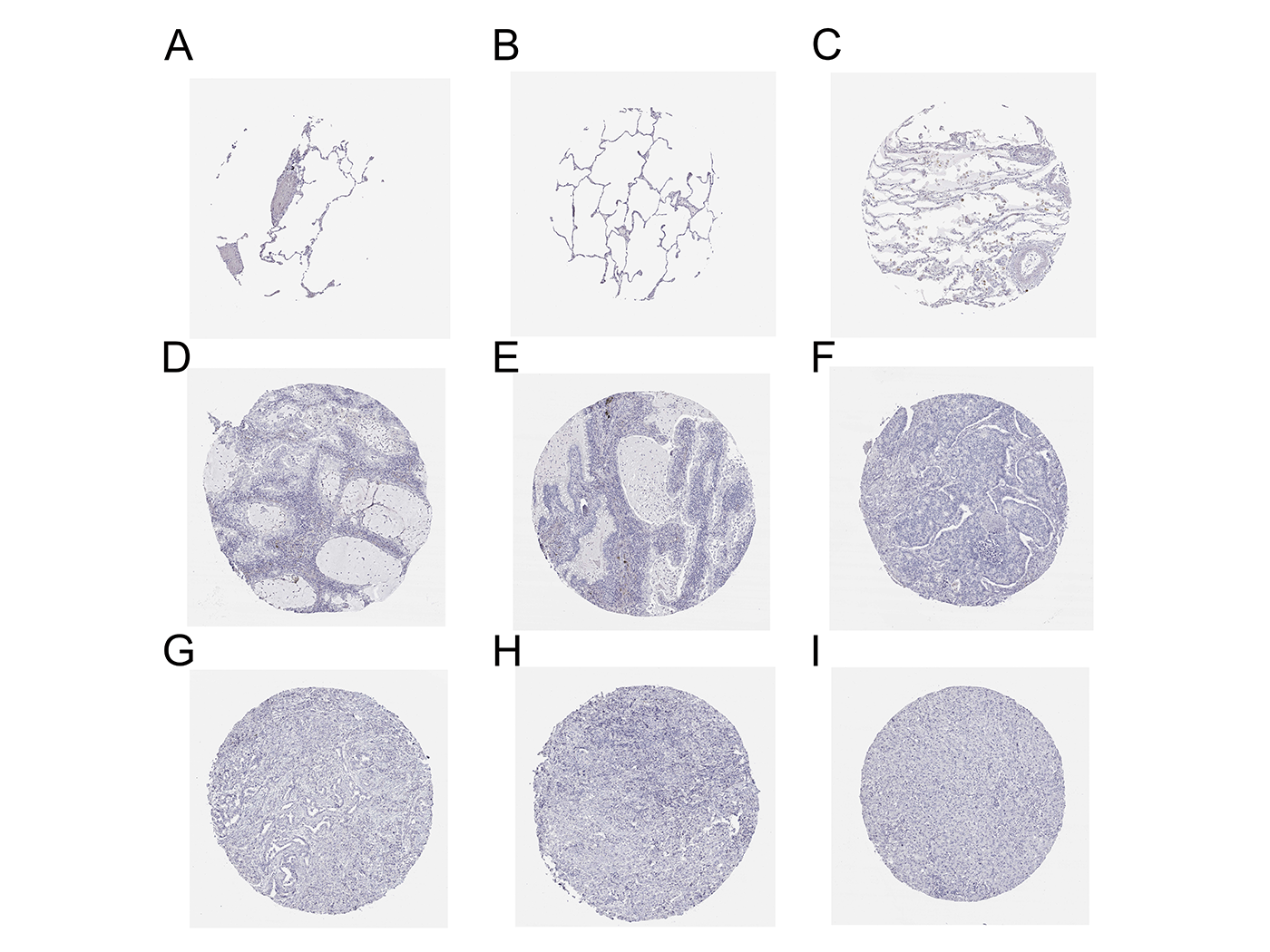

Supplement: Supplementary file 1 [file DataSheet_1.zip › Image 4.TIF]

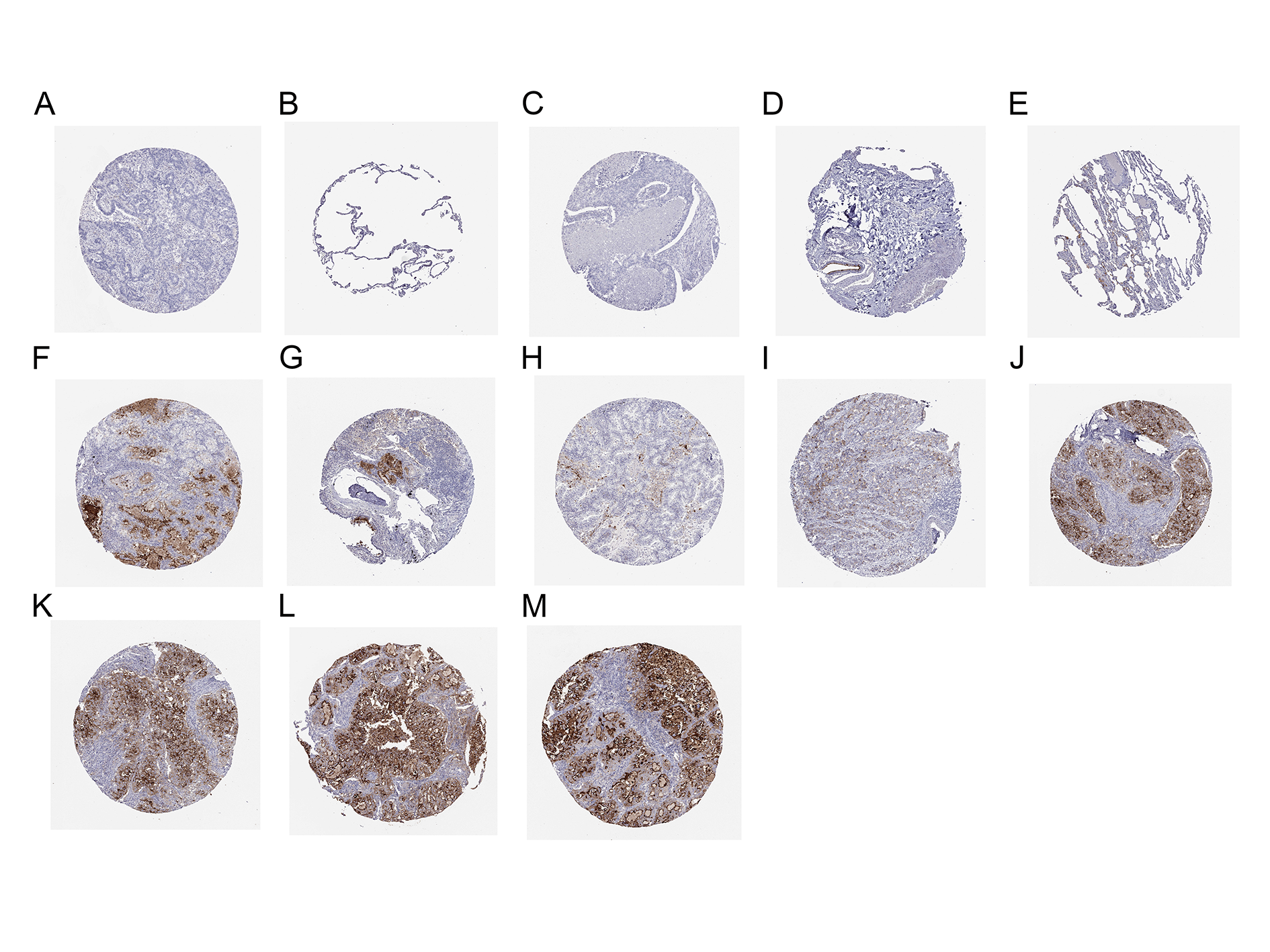

Supplement: Supplementary file 1 [file DataSheet_1.zip › Image 5.TIF]

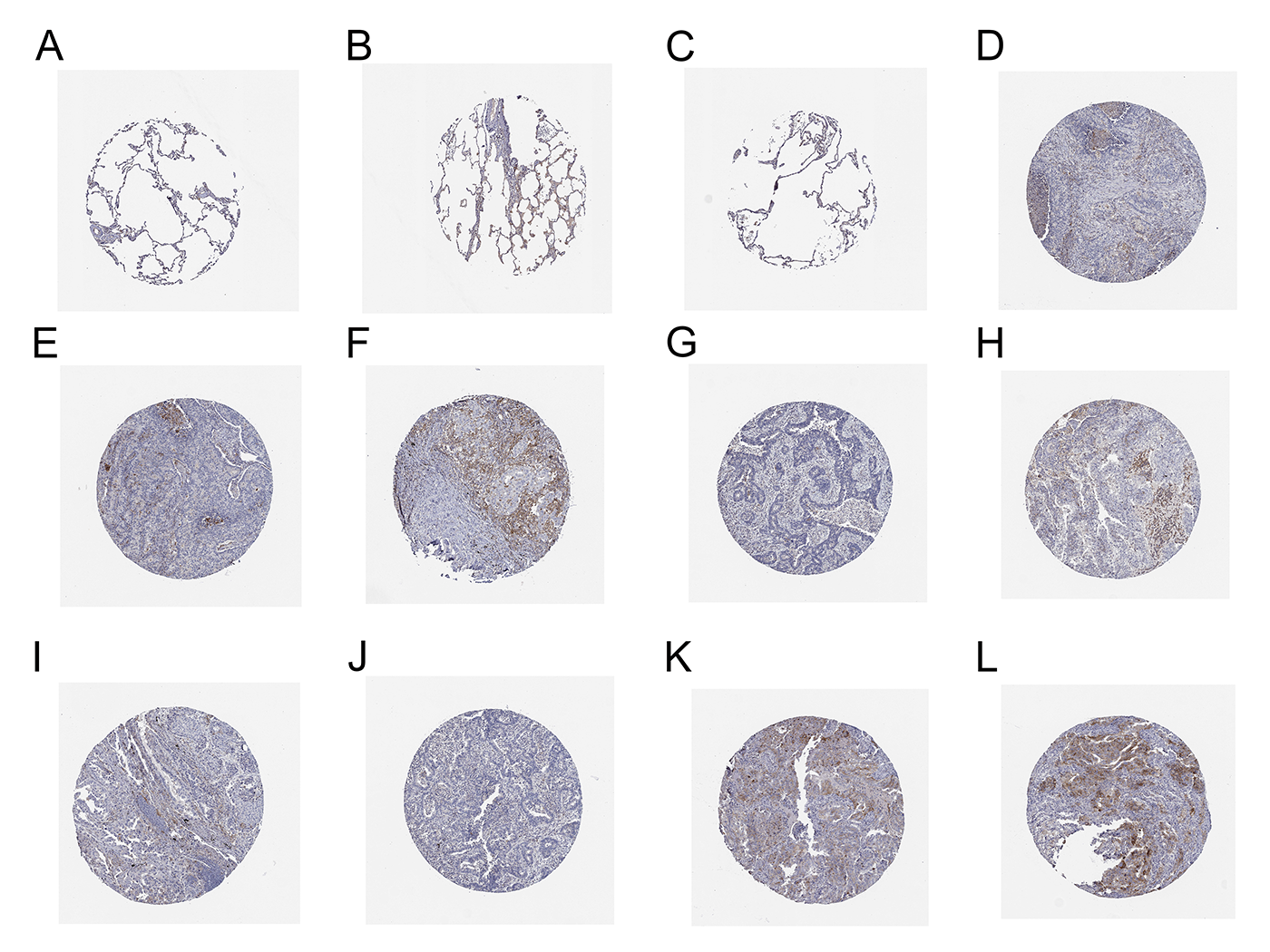

Supplement: Supplementary file 1 [file DataSheet_1.zip › Image 6.TIF]

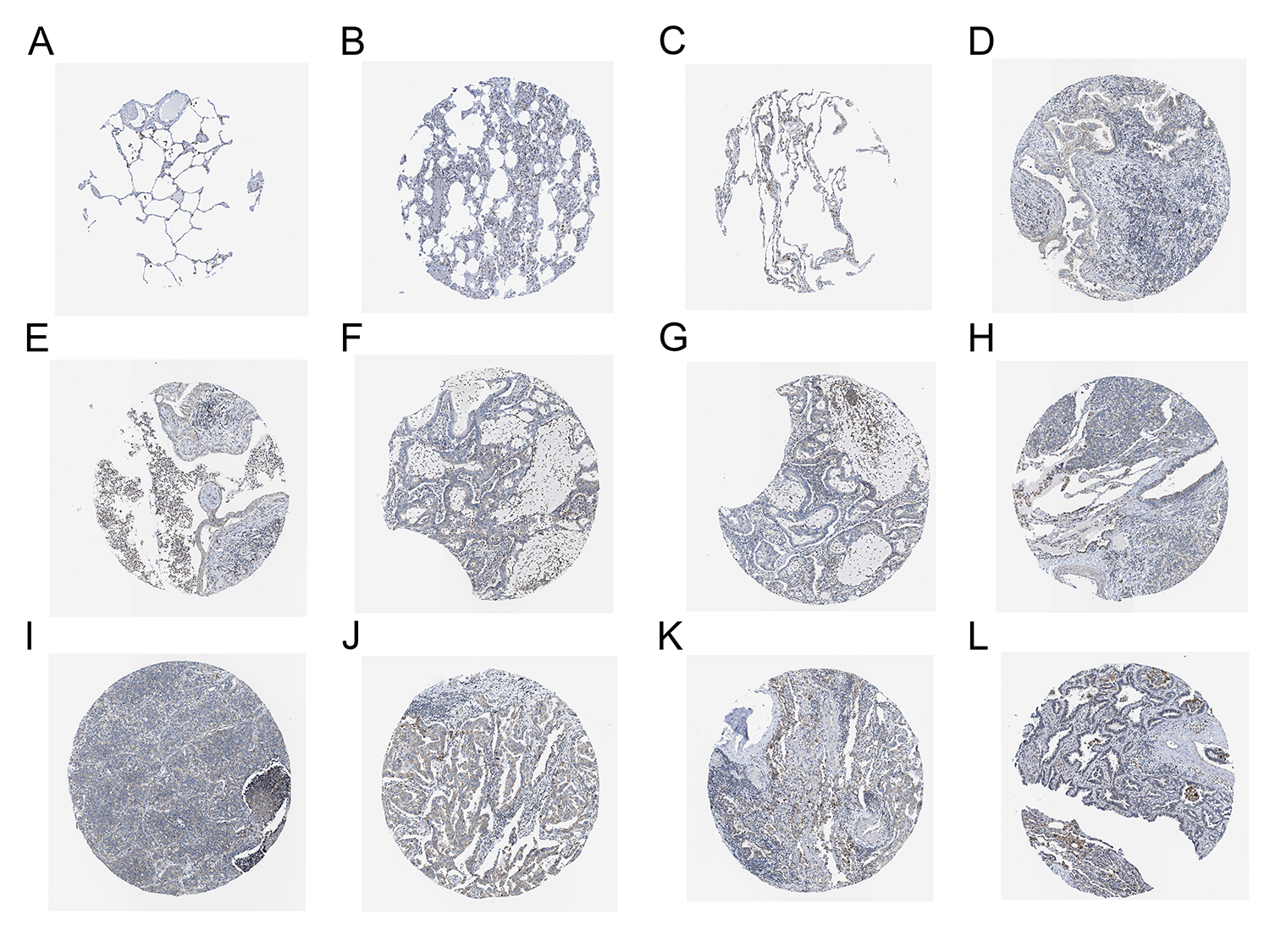

Supplement: Supplementary file 1 [file DataSheet_1.zip › Image 7.TIF]

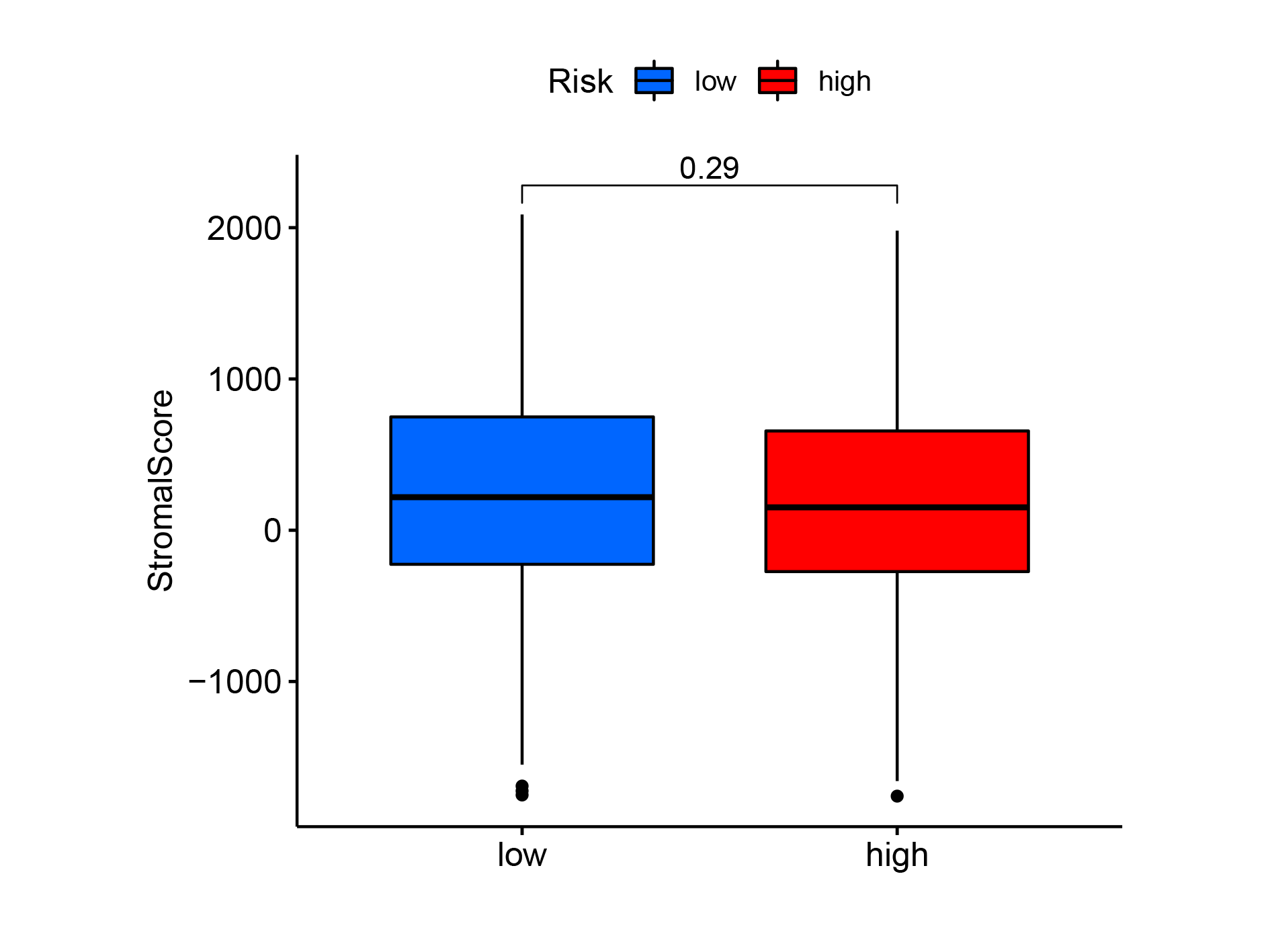

Supplement: Supplementary file 1 [file DataSheet_1.zip › Image 8.TIF]

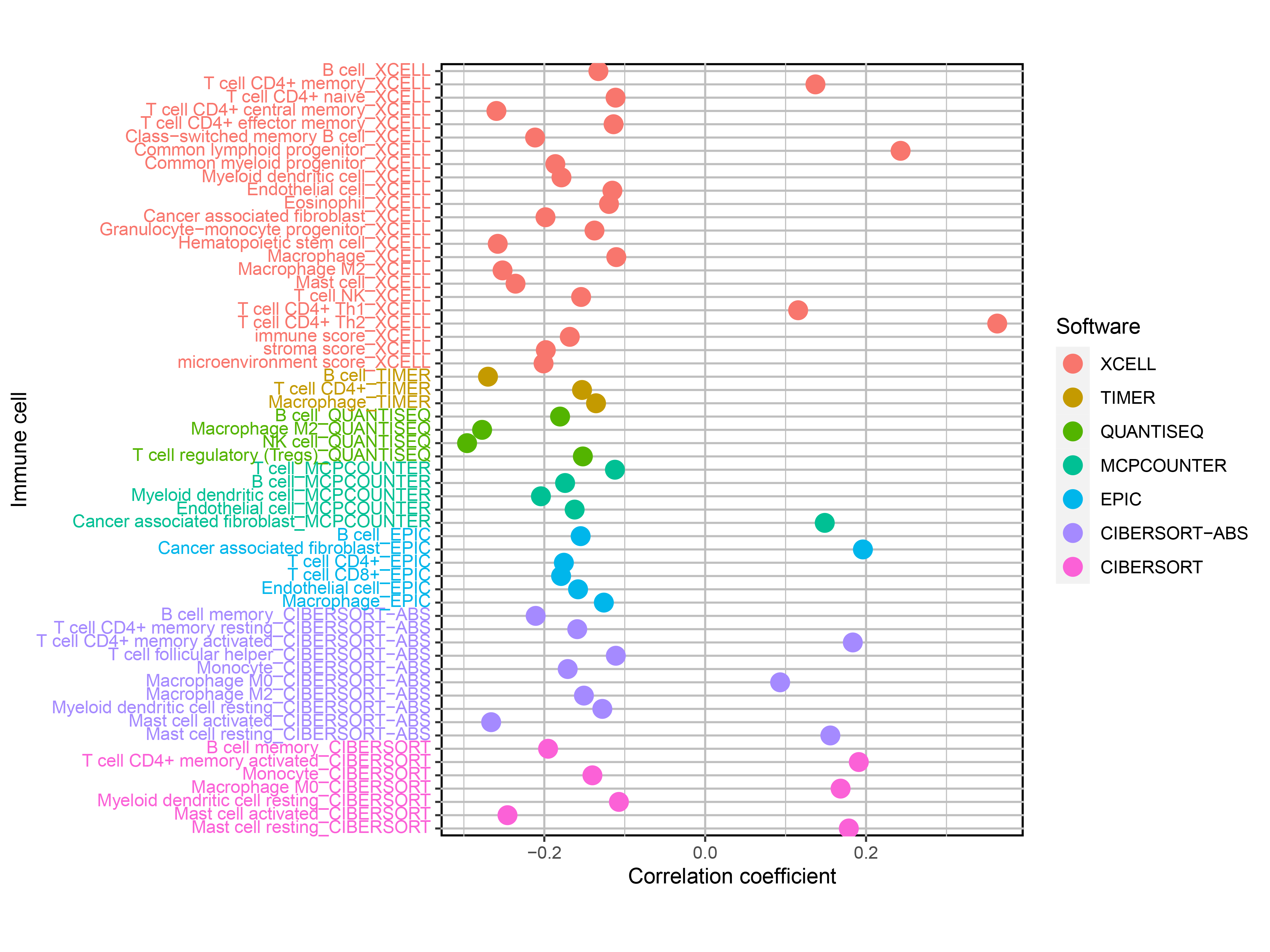

Supplement: Supplementary file 1 [file DataSheet_1.zip › Image 9.TIF]

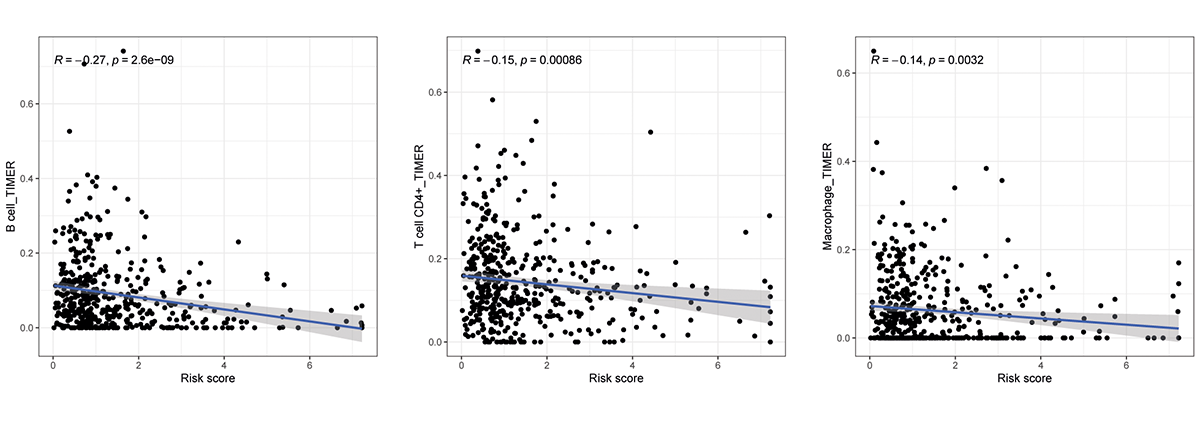

Supplement: Supplementary file 1 [file DataSheet_1.zip › Image 10.TIF]

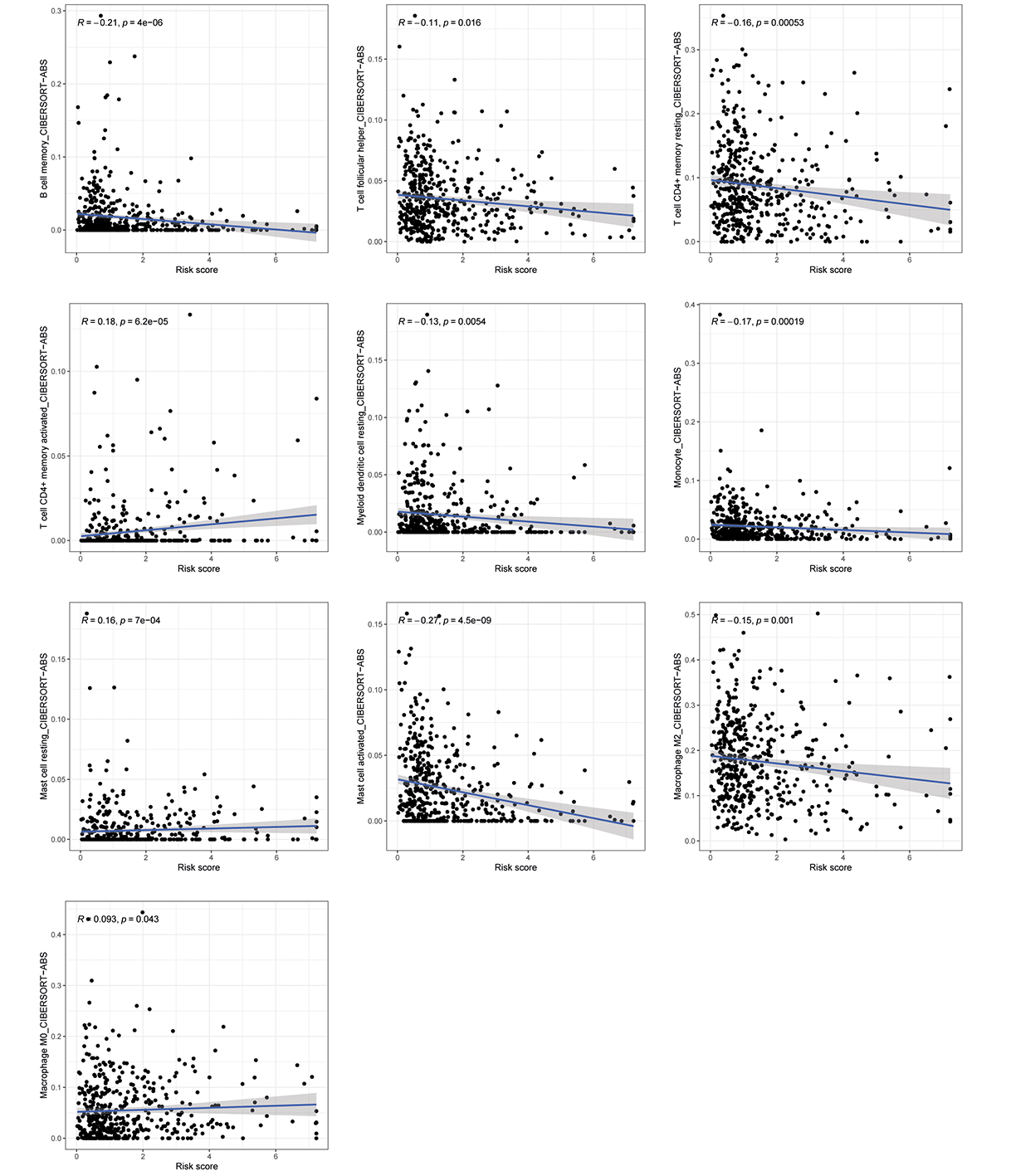

Supplement: Supplementary file 1 [file DataSheet_1.zip › Image 11.TIF]
