# Supplementary material for: Histone acetylation-related IncRNA: Potential biomarkers for predicting prognosis and immune response in lung adenocarcinoma, and distinguishing hot and cold tumours
Source: Front Immunol. 2023 Mar 17;14:1139599. doi: 10.3389/fimmu.2023.1139599 (PMC10064094; doi:10.3389/fimmu.2023.1139599)
Supplement: Supplementary file 1 [file DataSheet_1.zip › Table 2.DOC]

***Supplementary Figure Lend***

**Supplementary Figure 1**: Heat map of differential expression of 7 LNCRNAs between normal and Para cancerous tissues;

**Supplementary Figure 2**: C-index of risk score, age, gender stage；

**Supplementary Figure 3**：Immunohistochemical images of TP53 in normal tissues and tumor tissues; (A-C) Immunohistochemical image of TP53 in normal tissue，the corresponding immunohistochemical scores were 0, 0 and 0, respectively; (D-L) Immunohistochemical image of TP53 in tumor tissue, the corresponding immunohistochemical scores were 0, 0, 0, 0 , 0, 2 ,2 ,9, and 9, respectively；

**Supplementary Figure 4**：Immunohistochemical images of TTN in normal tissues and tumor tissues (A-C) Immunohistochemical image of TTN in normal tissue，the corresponding immunohistochemical scores were 0, 0 and 0, respectively;（D-I）Immunohistochemical images of TTN in tumor tissues, the corresponding immunohistochemical scores were 0, 0, 0, 0, 0 and 0, respectively;

**Supplementary Figure 5**: Immunohistochemical images of MUC16 in normal tissues and tumor tissues;（A-E）Immunohistochemical image of MUC16 in normal tissue, the corresponding immunohistochemical scores were 0, 0, 0, 0, 0 and 0, respectively;（E-M）Immunohistochemical image of MUC16 in tumor tissue, the corresponding immunohistochemical scores were 3, 3, 3, 3, 9, 9, 9 and 9, respectively;

**Supplementary Figure 6**: Immunohistochemical images of PD-L1 in normal tissues and tumor tissues;(A-C) Immunohistochemical image of PD-L1 in normal tissue, the corresponding immunohistochemical scores were 0, 0 and 0, respectively; (D-L) Immunohistochemical image of PD-L1 in tumor tissue, the corresponding immunohistochemical scores were 0, 0, 0, 0, 0, 0, 0, 3, and 3, respectively;

**Supplementary Figure 7**: Immunohistochemical images of PD-L2 in normal tissues and tumor tissues; (A-C) Immunohistochemical image of PD-L2 in normal tissue, the corresponding immunohistochemical scores were 2, 2 and 2, respectively; ;(D-L) Immunohistochemical image of PD-L2 in tumor tissue, the corresponding immunohistochemical scores were 4, 4, 4, 4, 6, 6, 6, 6, and 6;

**Supplementary Figure 8**: Differences in stromal score between high- and low-risk groups;

**Supplementary Figure 9**: Multiple platforms showed immune cell infiltration in high-risk group and low-risk group.

**Supplementary Figure 10**: TIMER platforms showed the correlation of immune cells in high and low risk groups；

**Supplementary Figure 11:** CIBERSORT-ABS platforms showed the correlation of immune cells in high and low risk groups；

**Supplementary Figure 12:** MCPCOUNTER platforms showed the correlation of immune cells in high and low risk groups；

**Supplementary Figure 13:** XCELL platforms showed the correlation of immune cells in high and low risk groups；

**Supplementary Figure 14:** EPIC algorithm showed the correlation of immune cells in high and low risk groups；

**Supplementary Figure 15:** CIBERSORT algorithm showed the correlation of immune cells in high and low risk groups；

**Supplementary Figure 16:** QUANTISEQ algorithm showed the correlation of immune cells in high and low risk groups；

**Supplementary Figure 17**: Tide score of Test group.

**Supplementary Figure 18:**Unsupervised consensus clustering of histone acetylation-related lncRNA in LUAD patients. (A-D) Consensus matrices of the training cohort for K = 1, 2,4,5. (E) Cumulative distribution function (CDF). (F) Relative change of area under CDF curve.
